# VarGoats project: a dataset of 1159 whole-genome sequences to dissect *Capra hircus* global diversity

**DOI:** 10.1186/s12711-021-00659-6

**Published:** 2021-11-08

**Authors:** Laure Denoyelle, Estelle Talouarn, Philippe Bardou, Licia Colli, Adriana Alberti, Coralie Danchin, Marcello Del Corvo, Stéfan Engelen, Céline Orvain, Isabelle Palhière, Rachel Rupp, Julien Sarry, Mazdak Salavati, Marcel Amills, Emily Clark, Paola Crepaldi, Thomas Faraut, Clet Wandui Masiga, François Pompanon, Benjamin D. Rosen, Alessandra Stella, Curtis P. Van Tassell, Gwenola Tosser-Klopp, The VarGoats Consortium, James Kijas, Bernt Guldbrandtsen, Juha Kantanen, Dylan Duby, Pierre Martin, Coralie Danchin, Delphine Duclos, Daniel Allain, Rémy Arquet, Nathalie Mandonnet, Michel Naves, Isabelle Palhière, Rachel Rupp, CABRICOOP breeders, François Pompanon, Hamid R. Rezaei, Sean Carolan, Maeve Foran, Alessandra Stella, Paolo Ajmone-Marsan, Licia Colli, Alessandra Crisà, Donata Marletta, Paola Crepaldi, Michele Ottino, Ettore Randi, Badr Benjelloun, Hans Lenstra, Muhammad Moaeen-ud-Din, Jim Reecy, Felix Goyache, Isabel Alvarez, Marcel Amills, Armand Sànchez, Juan Capote, Jordi Jordana, Agueda Pons, Amparo Martínez, Antonio Molina, Benjamin Rosen, Carina Visser, Cord Drögemüller, Gordon Luikart, Clet Wandui Masiga, Denis Fidalis Mujibi, Hassan Ally Mruttu, Timothy Gondwe, Joseph Sikosana, Maria Taela Da Gloria, Oyekan Nash

**Affiliations:** 1 GenPhySE, Université de Toulouse, INRAE, ENVT, 31326 Castanet-Tolosan, France; 2 Université Grenoble Alpes, Université Savoie Mont Blanc, CNRS, LECA, 38000 Grenoble, France; 3 Sigenae, INRAE, 31326 Castanet-Tolosan, France; 4 Dipartimento Di Scienze Animali, Della Nutrizione E Degli Alimenti, BioDNA Centro Di Ricerca Sulla Biodiversità E Sul DNA Antico, Facoltà Di Scienze Agrarie, Alimentari E Ambientali, Università Cattolica del Sacro Cuore, Milan, Italy; 5 Génomique Métabolique, Genoscope, Institut François Jacob, CEA, CNRS, Univ Evry, Université Paris-Saclay, 91057 Evry, France; 6 Institut de L’Elevage, Maison Nationale Des Eleveurs, 149 Rue de Bercy, 75595 Paris cedex 12, France; 7 The Roslin Institute and R(D)SVS, University of Edinburgh, Easter Bush Campus, Edinburgh, EH25 9RG UK; 8 Centre for Tropical Livestock Genetics and Health (CTLGH), Easter Bush Campus, Edinburgh, EH25 9RG UK; 9 Centre for Research in Agricultural Genomics (CRAG), CSIC-IRTA-UAB-UB, Universitat Autònoma de Barcelona, 08193 Bellaterra, Spain; 10 Depth. Agricultural and Environmental Sciences-Production, Landscape, Agroenergy, University of Milan, Milan, Italy; 11 Tropical Institute of Development Innovations (TRIDI), P O Box 23158, Kampala, Uganda; 12 Animal Genomics and Improvement Laboratory, BARC, USDA-ARS, Beltsville, MD 20705 USA; 13 Istituto Di Biologia E Biotecnologia Agraria, Consiglio Nazionale Delle Ricerche, Milan, Italy

## Abstract

**Background:**

Since their domestication 10,500 years ago, goat populations with distinctive genetic backgrounds have adapted to a broad variety of environments and breeding conditions. The VarGoats project is an international 1000-genome resequencing program designed to understand the consequences of domestication and breeding on the genetic diversity of domestic goats and to elucidate how speciation and hybridization have modeled the genomes of a set of species representative of the genus *Capra*.

**Findings:**

A dataset comprising 652 sequenced goats and 507 public goat sequences, including 35 animals representing eight wild species, has been collected worldwide. We identified 74,274,427 single nucleotide polymorphisms (SNPs) and 13,607,850 insertion-deletions (InDels) by aligning these sequences to the latest version of the goat reference genome (ARS1). A Neighbor-joining tree based on Reynolds genetic distances showed that goats from Africa, Asia and Europe tend to group into independent clusters. Because goat breeds from Oceania and Caribbean (Creole) all derive from imported animals, they are distributed along the tree according to their ancestral geographic origin.

**Conclusions:**

We report on an unprecedented international effort to characterize the genome-wide diversity of domestic goats. This large range of sequenced individuals represents a unique opportunity to ascertain how the demographic and selection processes associated with post-domestication history have shaped the diversity of this species. Data generated for the project will also be extremely useful to identify deleterious mutations and polymorphisms with causal effects on complex traits, and thus will contribute to new knowledge that could be used in genomic prediction and genome-wide association studies.

**Supplementary Information:**

The online version contains supplementary material available at 10.1186/s12711-021-00659-6.

## Context

Goats (*Capra hircus*) were domesticated around 10,500 years ago in the Fertile Crescent [1]. After their dispersion from their center of domestication, goats have undergone intense adaptation and occupy diverse agroecological zones around the world. As a result of selection, different breeds and lines of goats are now specialized for production of milk, meat, fiber [2], and also fuel or fertilizer from manure, thus goats play an important role in the livestock sector around the world. According to FAOSTAT (www.fao.org/faostat/en), the global goat population has increased by 38% since 1994, reaching over one billion heads in 2017. The global goat population is the third largest among ruminant production species.

In 2010, the first goat reference genome was sequenced and assembled [3], and the International Goat Genome Consortium (IGGC) was created to support the development of genomic tools for studying the genetic variation of domestic goats. The next major step was the development of the GoatSNP50 BeadChip in 2014 [4], a 50 k single nucleotide polymorphism (SNP) panel. The GoatSNP50 chip enabled the discovery of quantitative trait locus (QTL) through genome-wide association studies [5–7] and helped to initiate genomic selection in goats [8, 9]. The GoatSNP50 chip also facilitated international collaborations because it generated data through unrelated studies and different laboratories located across the globe that were directly comparable and could be easily merged. The AdaptMap project [10–16] was one of these collaborations that compiled goat genotypes from across the world and explored their genetic diversity. Although the AdaptMap dataset collected data from 4653 animals across 148 populations and 35 countries [14], it was limited to a subset of countries, and did not fully represent the variability of the *Capra* species worldwide. Indeed, wild goat species other than the bezoar (*Capra aegagrus*) were not investigated. Moreover, data generated from SNP chips are known to be distorted by ascertainment bias [17], a limitation that can be overcome by carefully filtering whole-genome sequencing data of sufficient depth [18].

In this paper, we report an international resequencing effort, the VarGoats project, that has generated data from 652 novel goat genomes combined with 507 existing genome sequences retrieved from public databases. We describe how this comprehensive dataset of 1159 genomes was obtained and characterized, and also present a perspective about the genetic relationships between domestic goat breeds on a worldwide scale.

## Findings

### Data description

#### Selection of individual goats

Animals sequenced in the VarGoats project were selected such that they represent the global genetic diversity of goats. First, based on the analysis of Colli et al. [15], 468 animals included in the AdaptMap dataset were selected to cover each of the 19 major gene pools determined by the Admixture software, except that from North America. Whenever possible, four to five breeds were selected within each group to ensure a fair representation of within-gene pool diversity. Eleven of the individuals that were included in the AdaptMap genotyped population were not included in the final dataset (https://datadryad.org/stash/dataset/doi:10.5061/dryad.v8g21pt), from which we extracted filtered genotyping data, thus genotypes were available for 457 individuals. To maximize the diversity of goats represented in this population, in addition to the 468 animals selected from the AdaptMap population, 184 other animals were selected to epitomise individual breeds and lineages and to allow investigation of adaptation to unique geographic or ecological niches. These 184 animals were selected from samples submitted by consortium members. After collecting these 652 (468 + 184) individuals that were sequenced within the VarGoats project, we retrieved 217 additional sequences through the Omics Discovery Index from NextGen consortium projects (PRJEB3134, PRJEB3135, PRJEB3136, PRJEB4371, PRJEB5166, and PRJEB5900), and 290 sequences from public sequence data repositories (sequences extracted from NCBI 7 Feb 2019). Thus, the VarGoats dataset included 1159 goat whole sequenced genomes.

**Fig. 1:**
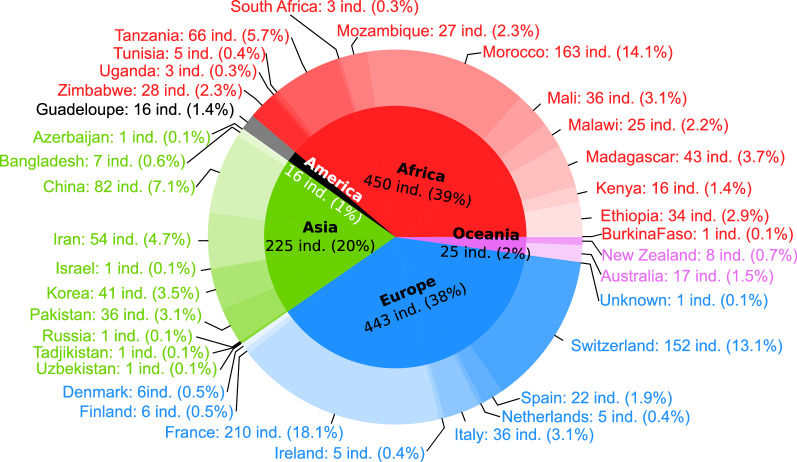
Geographical distribution of the 1159 sampled *Capra* individuals included in the dataset. The geographical origin of each sequenced animal is indicated both for the country (exterior circle) and the continent (interior circle). For each location, the number of sequenced individuals and their percentage in the overall dataset is indicated

Besides our efforts to maximize the amount of diversity represented in the VarGoat dataset, part of the sequencing effort aimed at addressing specific research questions. For example, since one of our objectives was to better understand the genetic variation underpinning commercial dairy breeds, more individuals from the Alpine and Saanen breeds than from other breeds were selected for sequencing. It should also be noted that several of the sequenced individuals are closely related. Among the 1159 sequenced animals, a vast majority (1124) correspond to domestic goats (*C. hircus*), which represent a range of breeds from Africa (450: 39%), Europe (443, 38%), Asia (225: 20%), Oceania (25: 2%) and the Caribbean (16: 1%). The geographical distribution of the investigated populations is shown in Fig. 1. The remaining 35 (3%) individuals represent wild *Capra* species, including *C. aegagrus*, *Capra caucasica*, *Capra cylindricornis*, *Capra falconeri*, *Capra ibex*, *Capra nubiana*, *Capra pyrenaica*, and *Capra sibirica*.

#### Code used to identify the animals

A unique identification code (original ID) defined each individual. For all sequenced animals, the ID was formatted as CCSS-BBB-NNNN: where CC corresponds to the country of origin based on the ISO 3166-1 alpha-2 codes, SS correspond to the species (CH for *C. hircus*, CA for *C. aegagrus,* etc.), BBB is the three character breed abbreviation (UNK for unknown), and NNNN is a four-digit number (with leading zeros) that is assigned sequentially within each combination of source country, species, and breed and that uniquely identifies each individual. Breed and country codes are in Additional file 1: Table S1. For the NextGen data, we used the sample’s alias as the original ID, and the run’s accession number as the original ID for the remaining public data.

An alternative ID, a ‘working name’, was assigned for each individual to facilitate the interpretation of the results for animals from unknown breeds. The working name format is similar to the original ID and is composed in the same way, except for a duplication of the country code as shown: CCSS-BBB_CC-NNNN.

For public data, information was extracted from BioSample (NCBI, https://www.ncbi.nlm.nih.gov/biosample/) in order to determine the sex, breed and geographic origin of each animal. For the VarGoats samples, information was collected by VarGoats collaborators. Detailed information for each sequenced individual is in Additional file 2: Table S2.

#### Concordance rates of the 457 AdaptMap individuals and breed ascertainment of outliers

**Fig. 2:**
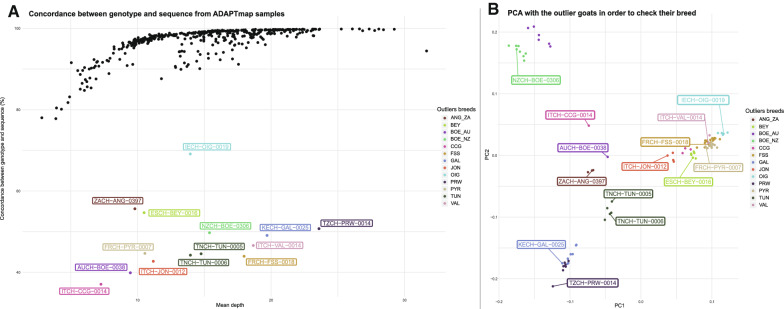
Analysis of the AdaptMap samples. **a** Concordance rates of the 457 AdaptMap individuals with 50 K chip and sequence data calculated on the basis of 44,691 common SNPs. **b** PCA to ascertain the breed of outlier animals

We extracted the 50 k genotypes for the 457 individuals with genotyping data from the AdaptMap project to detect potential sample mix-ups by checking the concordance rates (CR) between sequence variants and SNP array genotypes. Among the 46,654 SNPs genotyped in the AdaptMap project, 44,691 were identified in the VCF files from sequenced individuals. The distribution of CR clearly showed a disruption in this quality indicator at 70%, a value which we used as a threshold in the subsequent analyses (see Additional file 3: Figure S1). As shown in Fig. 2, low coverage sequences display lower CR values. For 14 individuals, although the sequencing depth was acceptable (between 7.2 and 23.6), a CR less than 70% was observed, which indicates a technical problem that leads to a lack of correspondence between the genotyped and sequenced samples (Fig. 2a.).

To check that the breed assignment indicated in the ID of these 14 animals was correct, we performed a principal component analysis (PCA) using the PLINK software version 1.9 [19] to compare the coordinates of these individuals with respect to all the animals belonging to those outlier breeds. The PCA was conducted on genotyping data of the autosomal SNPs from the VCF file. First of all, we removed SNPs with more than 5% of missing data. This filtering step yielded 1,890,194 SNPs which were pruned, using the indep-pairwise function (“--indep-pairwise 50 10 0.1”) in PLINK [19], i.e. SNPs in 50-SNP sliding windows with a step size of 10 SNPs displaying pairwise correlations between genotype allele counts greater than 0.1, were removed. This filtering step reduced the dataset to 667,949 SNPs which were used to assess population structure. Twelve of these 14 animals clustered with their breed counterparts (Fig. 2b), but for two individuals (AUCH-BOE-0038 and ITCH-CCG-0014) breed assignment could not be confirmed.

The 11 animals with missing genotypes, and therefore not confirmed, were marked in Additional file 2: Table S2 with a “1” in the fourth column, whereas the 14 animals with a concordance rate lower than 70% (Fig. 2a) were also marked with a “1” in the fifth column.

As recommended for any dataset, we advise users to perform a global analysis, such as a PCA or a population structure analysis based on the full dataset or on subsets to identify potential outliers before performing in-depth studies.

#### Breed information

The VarGoats dataset encompasses samples from eight wild species [(*C. aegagrus* (BEZ), *C. caucasica* (CAU), *C. pyrenaica* (CPY), *C. cylindricornis* (CYL), *C. falconeri* (FAL), *C. ibex* (IBX), *C. nubiana* (NUB), and *C. sibirica* (SIB)] and from 126 *C. hircus* breeds (see Additional file 1: Table S1). They are distributed as follows: 46 breeds from Africa (36%), 40 from Europe (32%), 34 from Asia (27%), 5 from Oceania (4%) and 1 from the Caribbean (1%). The dataset includes cosmopolitan breeds such as Alpine, Boer, and Saanen as well as local breeds that are unique to specific regions of the world.

**Fig. 3:**
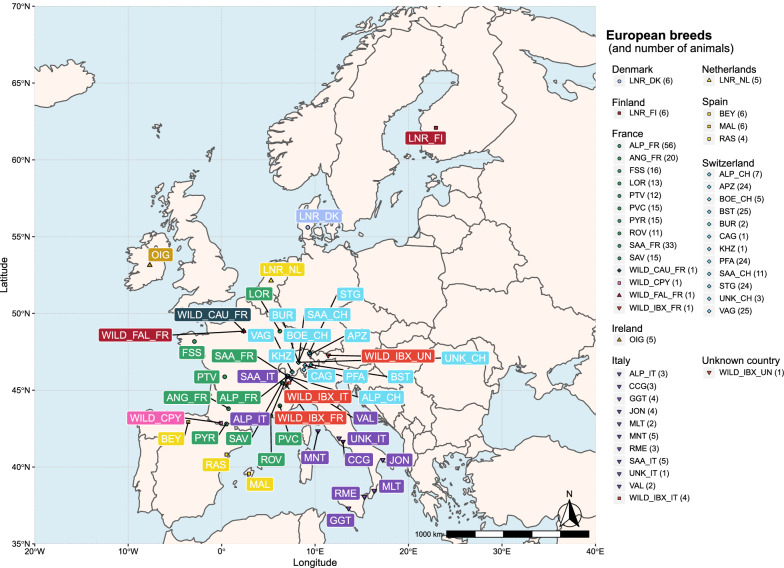
Geographic distribution of European breeds. Breeds are represented by three letters corresponding to the breed code (see Additional file 1: Table S1). If a breed is present in multiple countries, the breed code is followed by the country code (2 letters). Each combination of color and symbol corresponds to domestic goats in a single country. Wild goats are identified with a specific color and symbol

**Fig. 4:**
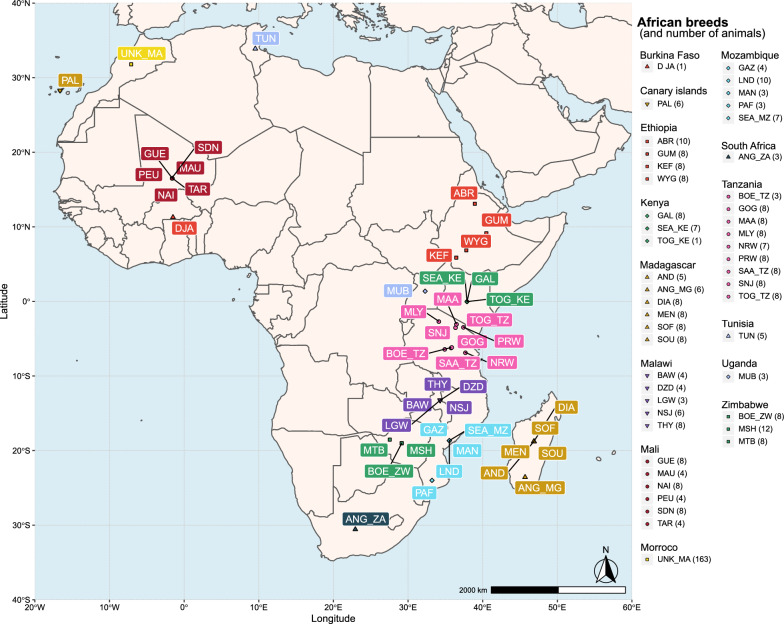
Geographic distribution of African breeds. Breeds are represented by three letters corresponding to the breed code (see Additional file 1: Table S1). If a breed is present in multiple countries, the breed code is followed by the country code (2 letters). Each combination of color and symbol corresponds to domestic goats in a single country

**Fig. 5:**
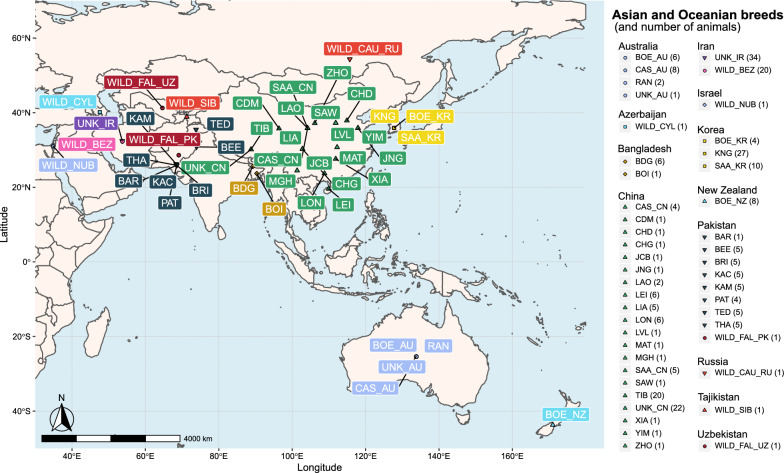
Geographic distribution of Asian and Oceanian breeds. Breeds are represented by three letters corresponding to the breed code (see Additional file 1: Table S1). If a breed is present in multiple countries, the breed code is followed by the country code (2 letters). Each combination of color and symbol corresponds to domestic goats in a single country. Wild goats are identified with a specific color and symbol

The geographical distribution of the breeds was determined based on a literature review (www.fao.org; www.racesdefrance.fr; http://eng.agraria.org/goat.htm; etc.), which allowed us to define more precisely how each breed was derived. In the absence of precise information, GPS coordinates of the country of origin (from the torop website) were assigned to each sample. Breed locations are represented for each continent in Figs. 3, 4 and 5, except for Creole, which is the only breed identified as Caribbean (specifically, West Indies).

### Library construction and sequencing

For all samples except those from African goats, two protocols were used for library preparation depending on the DNA extraction yield, i.e. 250 ng or much less. In all cases, sonication was performed with the E210 Covaris sonicator (Covaris, Inc., USA). When 250 ng of genomic DNA were available, the NEBNext DNA Modules Products (New England Biolabs, MA, USA) were used for end-repair, 3ʹ-adenylation and ligation of NextFlex DNA barcodes (Bioo Scientific Corporation, Saint-Marcel, France) of the DNA fragments. After two consecutive 1 × AMPure XP (Beckman Coulter France, Villepinte, France) clean-ups, the ligated fragments were amplified by 12 PCR cycles by using the Kapa Hifi Hotstart NGS library Amplification kit (Kapa Biosystems, Wilmington, MA, USA), followed by 0.6 × AMPure XP purification. When DNA extraction yielded DNA quantities much smaller than 250 ng, only 10 to 50 ng of genomic DNA were sonicated. Fragments were then end-repaired, 3ʹ-adenylated and NEXTflex DNA barcoded adapters were added by using NEBNext Ultra II DNA Library prep kit for Illumina (New England Biolabs, MA, USA). After two consecutive 1 × AMPure clean-ups, the ligated products were PCR-amplified with NEBNext® Ultra II Q5 Master Mix included in the kit, followed by 0.8 × AMPure XP purification.

All the libraries were subjected to size profile analysis, with an Agilent 2100 Bioanalyzer (Agilent Technologies, USA) and to qPCR quantification (MxPro, Agilent Technologies, USA). Libraries were sequenced using 151-bp length read chemistry in a paired-end flow cell on an Illumina HiSeq 4000 sequencer (Illumina, USA). On average, 120 million paired-end reads were obtained for each sample after clean up (12X expected genomic coverage).

Libraries corresponding to African goat samples were prepared by Edinburgh Genomics using the TruSeq Nano DNA High Throughput library preparation kit (Illumina, USA). The following preparation protocol was applied: 1 µg of genomic DNA was sheared to a mean fragment size of 450 bp using a Covaris LE220 focused-ultrasonicator (Covaris, Inc., USA). DNA fragments were then blunt-ended, A-tailed, size-selected and adapters were ligated to fragment ends according to the Illumina TruSeq PCR-free library preparation kit protocol. Insert size of the libraries was evaluated using a PerkinElmer LapChip GX Touch with an HT DNA 1 k/12 K/HI SENS LabChip and HT DNA HI SENS Reagent Kit (PerkinElmer, Inc., MA, USA). Final library concentration was calculated by qPCR using a Roche LightCycler 480 (Roche Molecular Systems, Inc., Switzerland) and a Kapa Illumina Library Quantification kit and Standards. Then, libraries were normalized to a loading concentration of 150 nM. All library processing steps were carried out on Hamilton MicroLab STAR (Hamilton Company, NV, USA) liquid handling robots coupled to BaseSpace Clarity LIMS X Edition (Illumina, CA, USA). Libraries were loaded into a HiSeq X Flow cell v2.5 and clustered using an Illumina cBot2 Illumina, CA, USA). Clustered flow cells were sequenced at a 15X coverage using a HiSeq X Ten Reagent kit v2.5.

Raw data were filtered to remove clusters that have too ‘much intensity’ corresponding to bases other than the called base. By default, the purity of the signal from each cluster is examined over the first 25 amplification cycles and calculated as Chastity = Highest_Intensity/(Highest_Intensity + Next_Highest_Intensity) for each cycle. The default filtering implemented at the base calling stage allows for at most one cycle with a low (< 0.6) Chastity score. Adapters and primers were removed from the sequence read and low-quality (< 20) nucleotides were trimmed from both ends. Sequences between the second unknown nucleotide (N) and the end of the read were also removed. Reads shorter than 30 nucleotides after trimming were discarded. Finally, the reads and their mates, that mapped to run quality control sequences (PhiX genome) were removed. These trimming steps were conducted using an in-house software based on the FastX package (http://www.genoscope.cns.fr/externe/fastxtend/).

### Data generation and preparation

#### Read alignment and variant calling

Sequence reads from each sample were processed using a pipeline based on the domain reference tools and the Genome Analysis Tool Kit—GATK (v3.6) best practices: the Burrows–Wheeler Aligner BWA software (v 0.7.15) for alignment [20], SAMtools (v1.6) for handling SAM/BAM file formats and calling variants [21], Picard tools (v2.1.1) for labelling duplicated reads, as well as GATK (v3.6) for insertion/deletion (InDel) realignment, base recalibration and calling variants [22], BCFtools (v1.6) for handling VCF/BCF formats, Freebayes (v1.1.0) for calling variants [23], and snpEff (v4.3t) for VCF annotation [24].

Reads were mapped to the latest ARS1 genome assembly (Genbank accession GCA_001704415.1) of *C. hircus* [25] using the BWA-MEM software with default parameters except for “-t 14 -M” and “-R” to add read groups. The SAM output files were converted to sorted BAM files using SAMtools.

Pre-processing steps (marking duplicates, InDel-based realignment and base quality score recalibration—(BQSR)) were done using the Picard-MarkDuplicates and GATK tools. The “known variants” file that is necessary for the BQSR step was computed on a subset of 13 goats (AUCH-CAS-0038, BFCH-DJA-0012, CHCH-BOE-0229, ESCH-PAL-0008, ESCH-RAS-0011, ETCH-ABR-0036, FICH-LNR-0122, FRCH-ALP-0030, FRCH-CRE-0014, FRCH-SAA-0032, ITCH-GGT-0026, MZCH-PAF-0003 and ZACH-ANG-0374), which represented 13 breeds chosen from the first 248 animals that were sequenced. These goat samples represented 11 of the 15 gene pools determined by Colli et al. [15] using the Admixture software [26]. We added one individual from the Creole breed (different from the Creole individuals genotyped in the AdaptMap dataset), as a representative of the American gene pool and one from an inbred breed (Palmera). Variants that fulfilled both of the following conditions were included in the “known variants” file: (1) variants that had at least six genotypes with at least one alternative allele ("snpSift filter 'countVariant() > 6'"); and (2) variants that were called by both Freebayes and GATK-HaplotypeCaller.

Variant calling for each individual was done with the GATK-HaplotypeCaller in ERC mode with a minimum read mapping quality of 30 and a minimum Phred-scaled confidence threshold of 30 (“-stand_call_conf 30.0 -mmq 30 -ERC GVCF -variant_index_type LINEAR -variant_index_parameter 128000”).

Due to the large number of samples, GVCF files were combined (CombineGVCFs) before the joint genotyping step (GenotypeGVCFs) to produce the raw VCF files by chromosome/scaffold.

#### Filtering process

A variant quality score recalibration (VQSR) step was performed on the raw VCF files. The same 13 goats that were used in variant calling were also used to establish training resource sets for VQSR calibration. Two training resources of true sites were built. The first set of variants included only the highest quality calls, with variants consistently identified with GATK, Mpileup [21] and Freebayes [23] (“known=false,training=true,truth=true,prior=15.0”). The second set of variants used the 60,000 SNPs selected by Tosser-Klopp et al. [4] to generate the set of SNPs included in the GoatSNP50 BeadChip (“known=false,training=true,truth=true,prior=12.0”). The training resource of non-true sites was built using the variants exclusively called by GATK (“known=false,training=true,truth=false,prior=10.0”). The last SNP resource, which was not used to train recalibration, was the dbSNP variant database provided by Ensembl version 95 (“known=true,training=false,truth=false,prior=2.0”). The variant call annotations (for SNPs and InDels) QD, DP, FS, MQRankSum, ReadPosRankSum, SOR and MQ (only for SNPs) were used for VariantRecalibrator. Additional file 4: Figures S2 and Additional file 5: Figure S3 show the pairwise comparisons of the annotations chosen in VQSR as described in the VQSR documentation. Based on the “snps. tranches” files (see Additional file 6: Figure S4 and Additional files 7 and 8), no false-positive variants were observed in the 99 tranche. Therefore, the 99.9 tranche was included to increase the sensitivity of variant discovery, because goats sampled for the VarGoats project included a wide range of breeds. We considered the highest tranche (99.9 to 100) as a false positive and excluded it. The remaining variants (SNPs and InDels) were then recalibrated at the truth sensitivity filter level (tranche) of 99.9.

In total, 129,043,954 SNPs were identified prior to VQSR filtering. Screening removed 14,799,331 (11.5%) loci, resulting in 114,244,623 variants passing the VQSR filter. Finally, only biallelic SNPs were retained with a GATK quality score greater than 100 and with at least two individuals carrying the alternative allele. The entire filtering process resulted in a high confidence set of 74,274,427 SNPs and 13,607,850 InDels (Table 1).

**Table 1:** Distribution of InDels and annotation of SNPs identified from sequences aligned to the ARS1 reference genome and using the GCF_001704415.1_ARS1_genomic.gff annotation file

CHI	InDel number	SNP number	Number of effects	Downstream	Exon	Intergenic	Intron	Splice acceptor	Splice donor	Splice region	Transcript	Upstream	UTR 3ʹ	UTR 5ʹ	Missense	Nonsense	Silent
1	818,757	4,445,377	12,032,012	458,743	66648	2,869,811	4,038,272	79	90	4866	4,076,351	453,685	51524	11943	21817	172	34232
2	681,619	3,704,710	11,025,471	500,555	86105	2,218,485	3,767,113	74	80	7110	3,886,499	493,018	53529	12903	27607	155	47266
3	582,990	3,219,852	9,723,073	683,259	99623	1,904,576	3,067,833	97	147	7253	3,210,217	676,123	57845	16100	34728	321	49798
4	612,055	3,356,824	10,217,296	402,190	63972	1,869,682	3,685,704	72	113	4137	3,747,652	394,011	40808	8955	21883	229	32329
5	589,455	3,254,170	10,416,604	608,369	90816	1,884,801	3,521,587	87	117	6961	3,628,991	604,367	56456	14052	28723	298	47717
6	628,940	3,423,894	9,712,976	356,081	49936	2,216,046	3,311,482	49	89	3940	3,376,966	352,520	36787	9080	16818	109	24491
7	534,662	2,892,084	9,828,378	636,813	159888	1,819,601	3,156,821	133	111	10127	3,347,312	631,783	51708	14081	63405	447	83894
8	558,356	3,063,425	9, 133,856	382,194	59762	1,965,117	3,107,183	63	69	4452	3,189,032	374,406	42124	9454	20793	209	30688
9	461,502	2,494,211	7,674,249	270,908	48106	1,572,204	2,714,425	138	46	3556	2,769,209	265,609	24246	5802	15956	125	23479
10	493,652	2,698,185	9,192,746	476,511	71317	1,511,953	3,242,782	52	96	5079	3,352,743	472,321	47467	12425	24247	221	37332
11	515,256	2,831,023	9,890,955	553,577	92208	1,724,793	3,395,147	92	136	7371	3,508,281	543,462	52415	13473	27137	241	51761
12	491,274	2,667,175	5,825,552	193,632	34358	1,765,985	1,782,658	33	48	2654	1,831,608	190,567	19095	4914	12736	158	17266
13	389,692	2,171,122	7,859,635	394,282	60365	1,203,702	2,832,529	77	91	4557	2,919,170	390,791	43851	10220	19308	201	32517
14	486,379	2,667,881	7,004,865	265,510	38581	1,771,173	2,298,648	41	52	3041	2,326,532	265,584	27828	7875	11194	96	19368
15	419,873	2,348,100	6,935,036	479,502	71589	1,493,439	2,136,669	53	84	4060	2,229,600	472,382	38348	9310	25431	347	33595
16	394,016	2,171,945	6,453,431	342,598	55559	1,256,466	2,169,250	56	60	4402	2,234,817	339,736	40836	9651	18793	155	29062
17	365,130	1,965,116	5,032,075	269,775	45016	1,219,875	1,562,271	38	91	3551	1,631,168	265,631	26028	8631	13371	108	24014
18	328,462	1,847,282	5,375,176	640,087	97496	1,052,854	1,380,659	112	152	6698	1,501,048	636,762	40352	18956	35046	523	45254
19	303,457	1,657,902	6,819,329	622,933	100340	808,006	2,215,917	76	65	7868	2,374,994	615,022	58429	15679	33492	286	57841
20	375,188	2,088,922	4,276,376	152,982	22276	1,434,583	1,232,290	25	25	1684	1,260,516	152,063	16729	3203	7060	39	11033
21	346,701	1,955,870	5,590,244	336,130	53444	1,238,872	1,791,260	100	118	3630	1,805,530	327,239	26317	7604	16845	199	24557
22	291,852	1,579,747	7,603,338	316,589	53838	756,258	3,024,571	51	52	4586	3,093,398	309,797	34524	9674	15493	103	29721
23	261,537	1,461,938	5,452,237	421,685	68240	852,094	1,785,250	47	91	4651	1,878,809	402,412	28019	10939	24936	313	34341
24	321,051	1,769,342	5,080,572	172,939	27021	1,192,978	1,727,081	12	27	2061	1,766,194	166,665	21637	3957	8023	62	14178
25	205,425	1,182,353	4,215,848	386,841	64350	533,065	1,350,422	47	88	4715	1,456,023	375,692	33162	11443	19585	144	32896
26	262,405	1,481,440	5,195,116	225,962	34955	839,548	1,896,442	41	74	2605	1,943,238	222,634	23591	6026	11600	139	16083
27	232,467	1,278,104	3,190,769	130,257	20512	844,522	1,003,995	32	31	1512	1,037,321	132,486	15547	4554	6764	43	10924
28	228,740	1,306,693	5,179,451	191,839	29690	693,876	2,002,455	47	27	2308	2,040,071	191,412	22278	5448	9736	66	14952
29	257,510	1,508,568	6,275,068	344,027	54609	844,994	2,303,306	65	78	3832	2,360,477	333,712	22868	7100	17940	133	28259
MT others	1,169,447	5,781,172	9,317,433	534,885	76267	4,875,855	1,611,366	133	149	4586	1,648,637	520,101	33765	11689	22371	370	31779
Total	13,607,850	74,274,427	221,529,167	11,751,655	1,896,887	46,235,214	73,115,388	2022	2497	137,853	75,432,404	11,571,993	1,088,113	295,141	632,838	6012	970,627

Number of InDels and SNPs identified per chromosome (CHI) and annotation information for SNPs identified

### Main features of the dataset

The GCF_001704415.1_ARS1_genomic.gff annotation file was used to annotate variants using the SnpEff software. Thus for each SNP, descriptive information is provided in the INFO field. The exhaustive description of the INFO field can be found in snpEff manual. For example, it contains a description of the position of the variant relative to a gene (upstream, downstream, exon, intron), if the variant hits a transcript (transcript), if it is located in an untranslated region (UTR 3ʹ, UTR 5ʹ), its location and effect regarding splicing (splice acceptor, splice donor, splice region), amino-acid changes (missense, nonsense, silent), etc. Table 1 summarizes the numbers and types of variants found on each chromosome for 14 annotations (downstream, exon, intergenic, intron, splice acceptor, splice donor, splice region, transcript, upstream, UTR 3ʹ, UTR 5ʹ, missense, nonsense, silent).

### Sex assignment

When known, the sex of each individual is provided in Additional file 2: Table S2. However, the sex of 128 animals (mostly public data) was not available and was inferred by using a pipeline described here. Based on each alignment file (BAM) for each goat, the depth of the sequencing reads per chromosome or scaffold was determined using the idxstat command of SAMtools. The read depth was standardized by chromosome or scaffold length as ([read number]/[chromosome or scaffold length] * 100). For the sex chromosomes, the mean standardized read depth was calculated as the standardized read depth for each scaffold weighted by the length of that scaffold. The mean standardized read depth for *C. hircus* (CHI) chromosome X (CHIX) is determined as the weighted standardized mean depth extracted from the scaffolds NW_017189516.1 and NW_017189517.1. The corresponding value for chromosome Y (CHIY) was calculated as the weighted standardized mean depth determined from scaffolds NW_017189563.1, NW_017189610.1, NW_017189618.1, NW_017189628.1, NW_017189685.1, NW_017189696.1, NW_017189885.1, NW_017189985.1, NW_017190040.1, NW_017190154.1, and NW_017195709.1. The ratio of the standardized mean read depth of the autosomes to CHIX was calculated using the weighted mean of the read depth across the autosomes. The expected ratio for females was 1 for CHIX. No reads from the Y scaffolds were expected. For males, alternatively, a two to one ratio is expected for read depth of autosomes to CHIX or CHIY. Examination of these two values for each animal (see Additional file 9: Figure S5) allowed us to determine a threshold that differentiates the two sexes. Individuals that had a mean standardized read depth of 25 or more from the Y scaffolds were considered to be males, and otherwise, they were assigned to the female category. This information is reported in Additional file 2: Table S2, in the “assigned sex” column. Sixteen animals showed discordances between recorded and inferred sex, which corresponds to a 1.6% error rate. Given that this percentage is quite low, we are confident that our method is accurate and we believe that such inconsistencies could be due to errors when declared sexes were recorded.

### Genetic differentiation

**Fig. 6:**
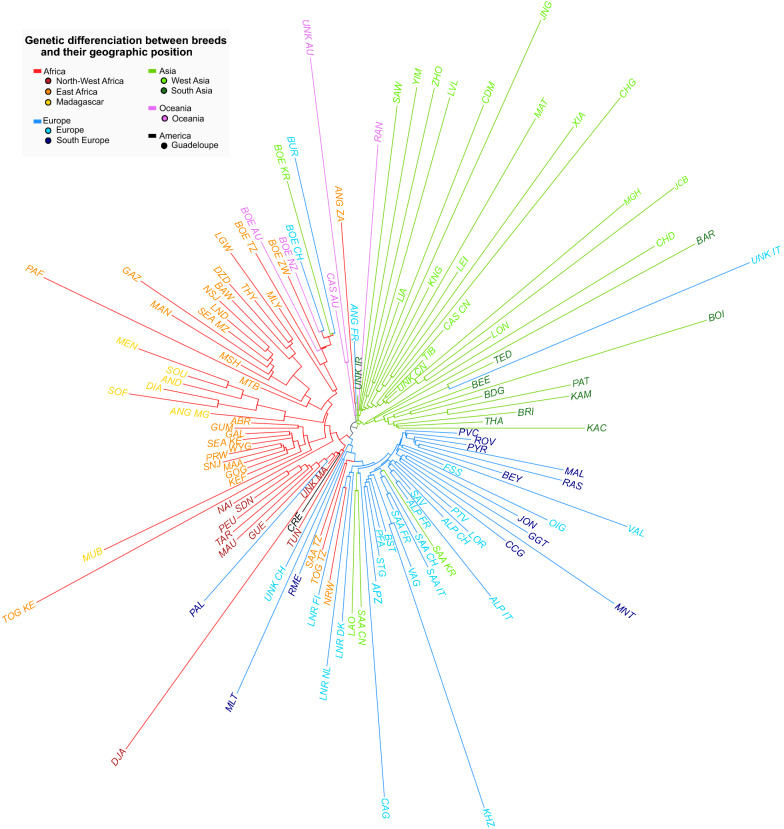
Neighbor-joining tree representing the genetic diversity of domestic goat populations analysed in the context of the VarGoats project. The three-letter breed code (see Additional file 1: Table S1) is used, followed by the two-letter country code if needed. Colors allow the identification of geographical origin

We conducted an exploratory analysis to evaluate the genetic differentiation among goat breeds. We included breeds that were represented by a single individual because we were interested in understanding the relationship among all the breeds in the overall dataset. To perform this genetic differentiation analysis, we used the thin-count function from PLINK [19] to extract a subset of random 100,000 SNPs from the reduced set of the 667,949 autosomal SNPs used in the PCA analysis (see “Selection of individual goats” section). Starting from this reduced dataset, a matrix of between-population Reynolds distances was calculated using the hapFLK v.1.3.0 program [27, 28] and then it was used to construct a Neighbor-Joining tree (Fig. 6). Since bezoar (*C. aegagrus*) is the wild species closest to domestic goats, it was used as an outgroup to root the tree.

In the neighbor-joining tree in Fig. 6, goats were grouped according to their ancestral geographic origin (Africa, Europe, Asia or Middle East). These results were consistent for the Oceanian breeds. Boer goats from Australia (BOE_AU) and New Zealand (BOE_NZ) clustered with other Boer populations sampled in Africa (BOE_TZ, BOE_ZW), Asia (BOE_KR), and Europe (BOE_CH). Similarly, Australian Cashmere (CAS_AU) goats clustered with Angora goats from France and South Africa because of their common Middle Eastern origin. A similar clustering pattern based on the ancestral geographic origin is also observed in transboundary breeds sampled in different countries (i.e., Alpine, Boer and Saanen).

The first branching among domestic goats separated the Asian breeds from the remaining populations. This branch carried the most ancestral cluster, which was mainly composed of breeds that originated in South-Western Asia (Iranian and Pakistani goats) and were thus geographically close to the center of domestication [1]. The second branch included a cluster composed of long-haired breeds (Angora and Cashmere goats). Two additional African and European clusters were observed. In each continental group, geographically coherent sub-clusters could be clearly distinguished (e.g. Northern and Southern Europe, North-Western Africa, Eastern Africa and Madagascar). Concerning the two animals with an ambiguous breed status, one (UNK_AU, ex AUCH-BOE-0038) grouped with long-haired goats and the other (UNK_IT, ex ITCH-CCG-0014) with Pakistani goats.

## Conclusions

This 1159-goat genome dataset (VarGoats dataset) provides an unprecedented resource for research studies on caprine genomics. We provide a detailed methodology for calling SNPs, detecting InDels and filtering these data, which has led to a dataset that was validated through a differentiation study. This allowed us to verify that samples were properly assigned to their corresponding breed and geographical origin (both for samples collected for this study and public data). This VarGoats dataset will enable an unprecedented view of the footprints of the natural and human-mediated evolutionary forces that have shaped the diversity of caprine genomes. The VarGoats Consortium is currently organized in working groups, with each team conducting detailed analyses of these data. Among the various topics that are under study are: (i) analyses of structural variations, (ii) population genetics analysis and population history domestication reconstruction, (iii) detection of selection and adaptation signatures, (iv) identification of loss of function alleles, and (v) comparison of the goat genomes with those of other ruminant species. These ongoing population genetics and genomic analyses of this dataset should lead to additional publications together with the public release of the variant datasets.

## Supplementary Information

**Additional file 1: Table S1.** Number of individuals per breed and country of origin. Distribution of sequenced individuals per breed and description of the abbreviations corresponding to each population.

**Additional file 2: Table S2.** Detailed information for each sequenced individual. Description of each individual (species, breed, country of origin, localization, sex, sample provider and details on its associated sequence).

**Additional file 3: Figure S1.** Distribution of the concordance rates. Concordance rates (CR) between sequence variants and 50 k genotypes for 457 individuals. The figure shows a clear disruption in the distribution of CR, thus we found it easy and relevant to discard the samples below a concordance rate of 70% between sequence and chip SNP data.

**Additional file 4: Figure S2.** Pairwise comparisons of the annotations chosen in VQSR for SNPs. Description: Modeling report generated by GATK VariantRecalibrator for every pairwise combination of annotations used (QD, DP, FS, MQRankSum, ReadPosRankSum, SOR and MQ), with a 2D projection of the Gaussian mixture model.

**Additional file 5: Figure S3.** Pairwise comparisons of the annotations chosen in VQSR for InDels. Description: Modeling report generated by GATK VariantRecalibrator for every pairwise combination of annotations used (QD, DP, FS, MQRankSum, ReadPosRankSum and SOR), with a 2D projection of the Gaussian mixture model.

**Additional file 6: Figure S4.** Tranche plot produced by VariantRecalibrator for SNPs. Description: Partition of the call sets into quality tranches. The tranches correspond to certain levels of sensitivity relative to the truth sets (the highest tranche corresponds to a high accuracy call set but with the lowest value of sensitivity).

**Additional file 7.** Tranche summary file produced by VariantRecalibrator for SNPs. File of comma-separated values for tranches 0 to 90, 90 to 99, 99 to 99.9 and 99.9 to 100.

**Additional file 8.** Tranche summary file produced by VariantRecalibrator for InDels. File of comma-separated values for tranches 0 to 90, 90 to 99, 99 to 99.9 and 99.9 to 100.

**Additional file 9: Figure S5.** Sex assignment for the 1159 goats sampled in VarGoats. Representation of the ratio of read number between the autosomes and the X chromosome and the read number on the Y chromosome for each animal to determine its sex. The horizontal bar corresponds to the threshold of 25 reads from the Y scaffold allowing to differentiate males from females.

## Data Availability

The VarGoats dataset is publicly available in the European Nucleotide Archive (ENA) as project number PRJEB37507, which includes fastq and sample description data for 266 animals under accession PRJEB31857, 337 animals under accession PRJEB37122, 29 animals under accession PRJEB37276 and 20 animals under PRJEB37208. The 290 additional sequences used in this article were retrieved from public databases and 217 from NextGen Consortium projects (PRJEB3134, PRJEB3135, PRJEB4371, PRJEB5166, PRJEB3136 and PRJEB5900 studies). Individual accession numbers are listed in Additional file 2: Table S2. Use of these data is regulated by a data sharing agreement which is available here: http://www.goatgenome.org/vargoats_agreement.html. This agreement states that it is mandatory to contact the VarGoats steering committee to discuss the utilization and inclusion of data generated by the VarGoats Consortium in any present or future publication. No publications can be generated from the Vargoats dataset until the main papers derived from this project are published in scientific journals. Scientists who have signed the agreement have access to 30 VCF files split by chromosome for SNPs and an additional VCF for InDels. They can also access the VCF used for the truth sets and the VQSR VCF file for SNPs.
