# Supplementary figures and images for: VarGoats project: a dataset of 1159 whole-genome sequences to dissect Capra hircus global diversity
Source: Genet Sel Evol. 2021 Nov 8;53:86. doi: 10.1186/s12711-021-00659-6 (PMC8573910; doi:10.1186/s12711-021-00659-6)

CR

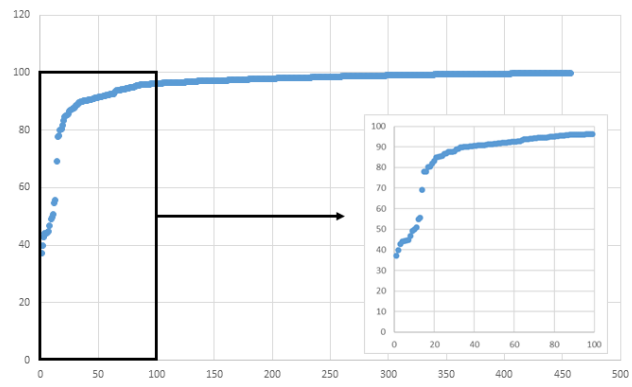

Supplement: Supplementary file 3 — Additional file 3: Figure S1. Distribution of the concordance rates. Concordance rates (CR) between sequence variants and 50 k genotypes for 457 individuals. The figure shows a clear disruption in the distribution of CR, thus we found it easy and relevant to discard the samples below a concordance rate of 70% between sequence and chip SNP data. [file 12711_2021_659_MOESM3_ESM.pdf]

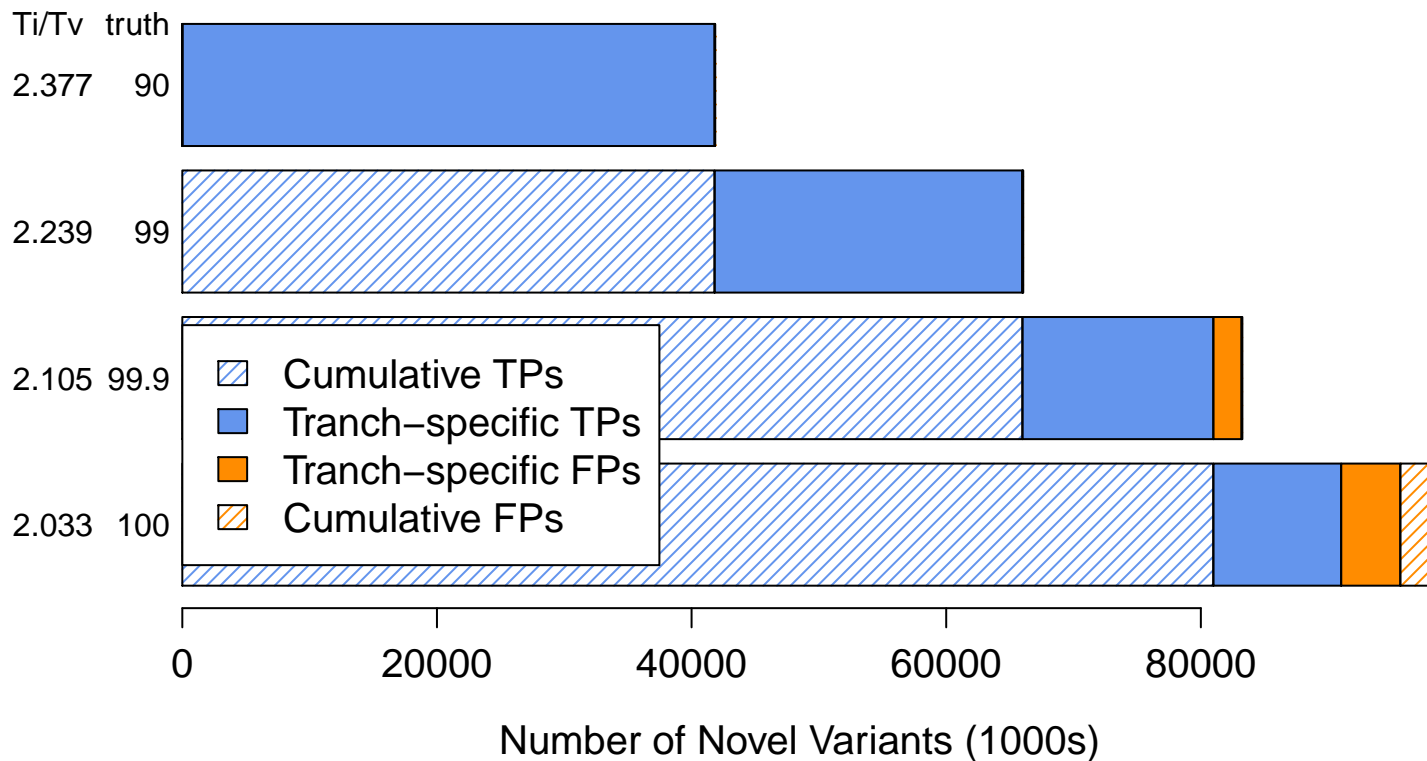

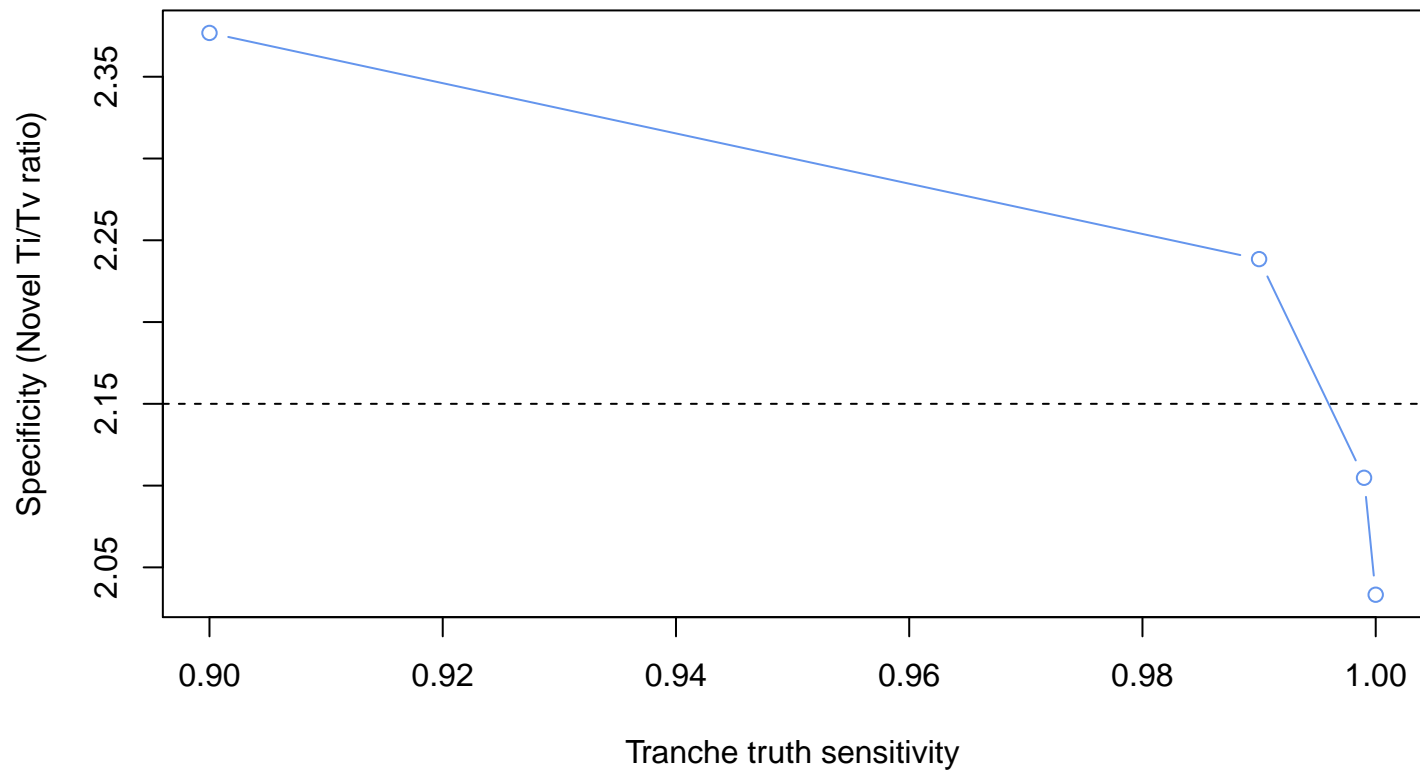

Supplement: Supplementary file 6 — Additional file 6: Figure S4. Tranche plot produced by VariantRecalibrator for SNPs. Description: Partition of the call sets into quality tranches. The tranches correspond to certain levels of sensitivity relative to the truth sets (the highest tranche corresponds to a high accuracy call set but with the lowest value of sensitivity). [file 12711_2021_659_MOESM6_ESM.pdf]

# Sex assignment

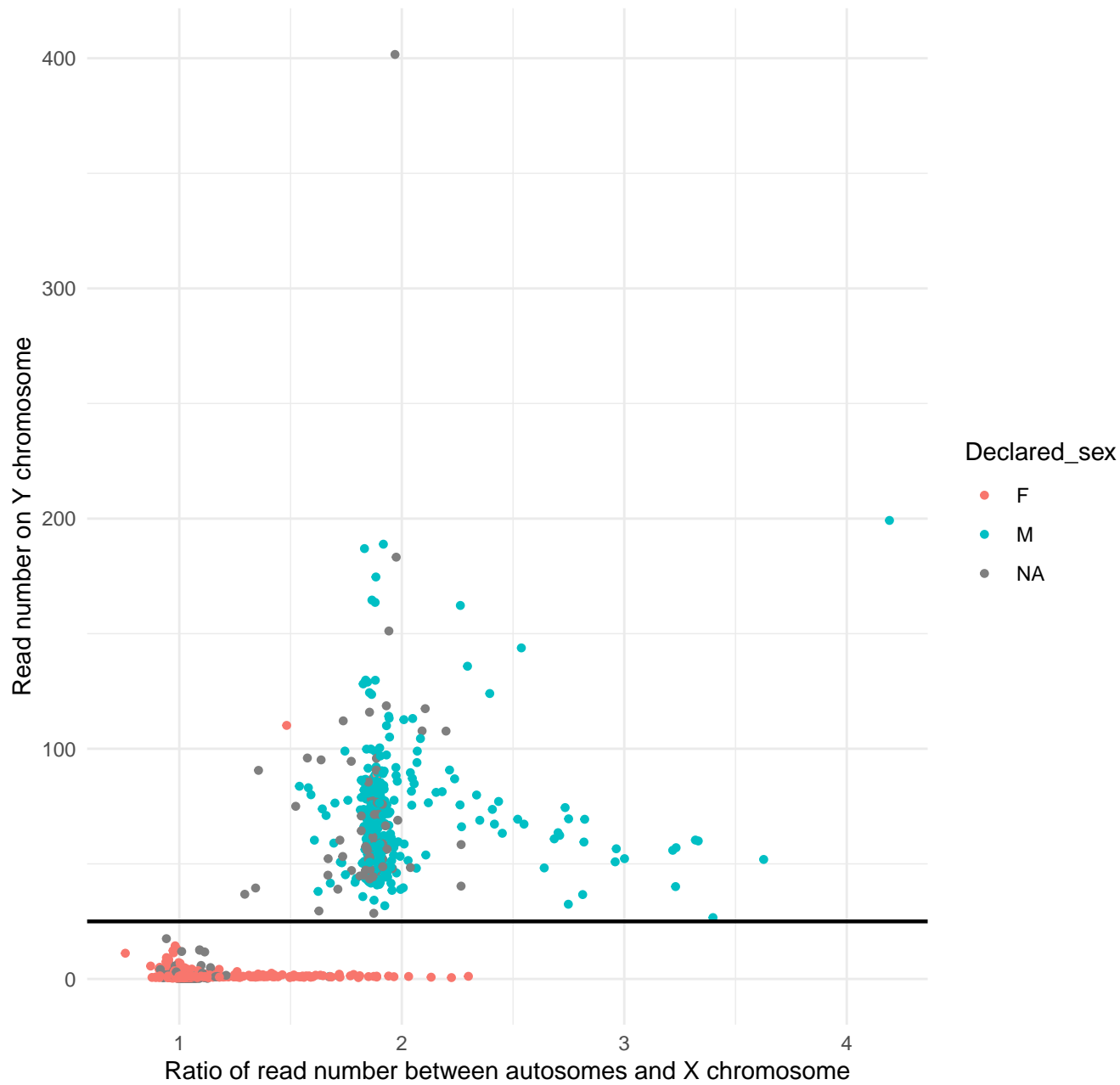

Supplement: Supplementary file 9 — Additional file 9: Figure S5. Sex assignment for the 1159 goats sampled in VarGoats. Representation of the ratio of read number between the autosomes and the X chromosome and the read number on the Y chromosome for each animal to determine its sex. The horizontal bar corresponds to the threshold of 25 reads from the Y scaffold allowing to differentiate males from females. [file 12711_2021_659_MOESM9_ESM.pdf]
