# Supplementary material for: VarGoats project: a dataset of 1159 whole-genome sequences to dissect Capra hircus global diversity
Source: Genet Sel Evol. 2021 Nov 8;53:86. doi: 10.1186/s12711-021-00659-6 (PMC8573910; doi:10.1186/s12711-021-00659-6)
Supplement: Supplementary file 4 — Additional file 4: Figure S2. Pairwise comparisons of the annotations chosen in VQSR for SNPs. Description: Modeling report generated by GATK VariantRecalibrator for every pairwise combination of annotations used (QD, DP, FS, MQRankSum, ReadPosRankSum, SOR and MQ), with a 2D projection of the Gaussian mixture model. [file 12711_2021_659_MOESM4_ESM.pdf]

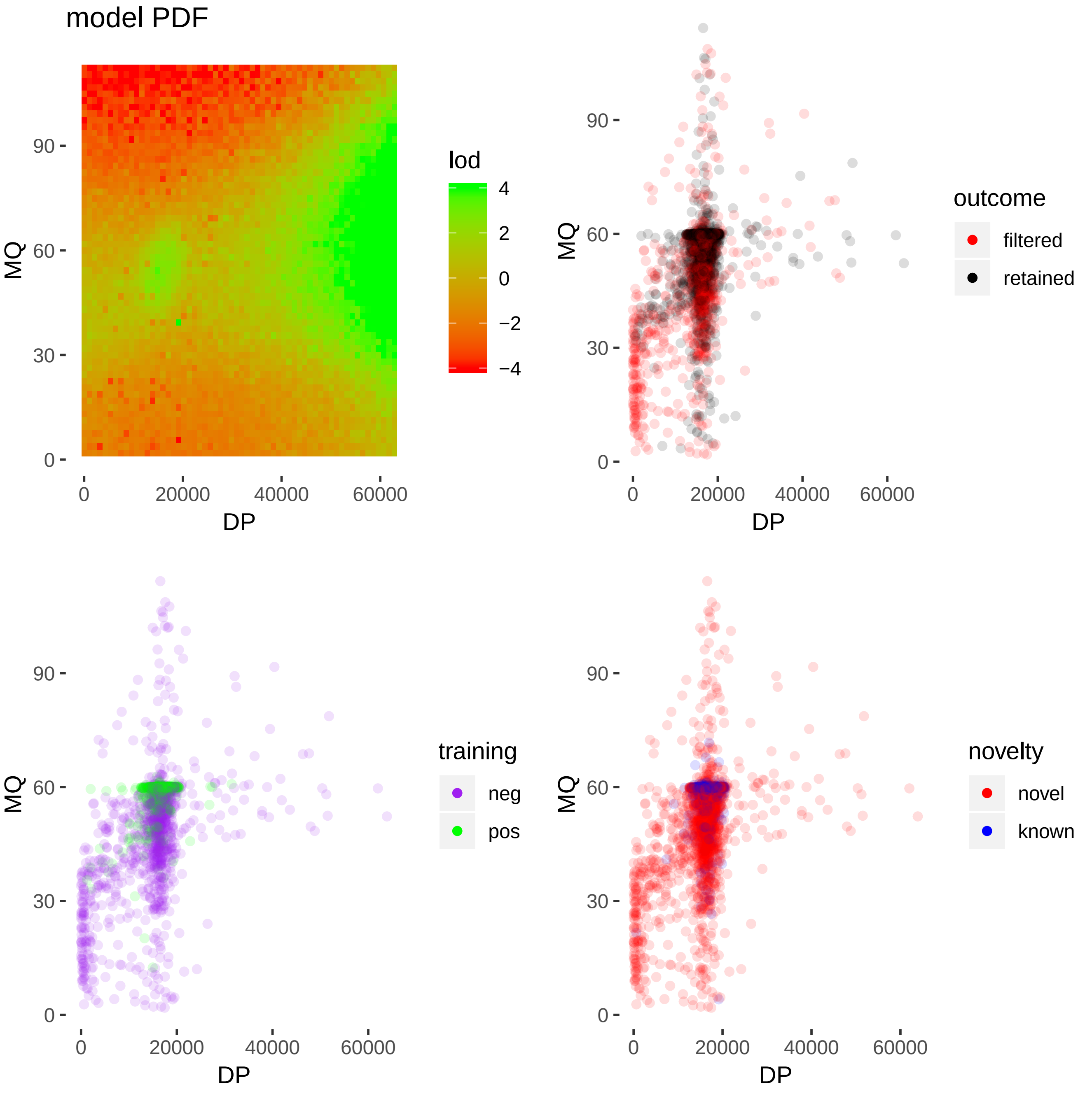

model PDF
MQ
DP
lod
4
2
0
−2
−4
outcome
filtered
retained
training
neg
pos
novelty
novel
known

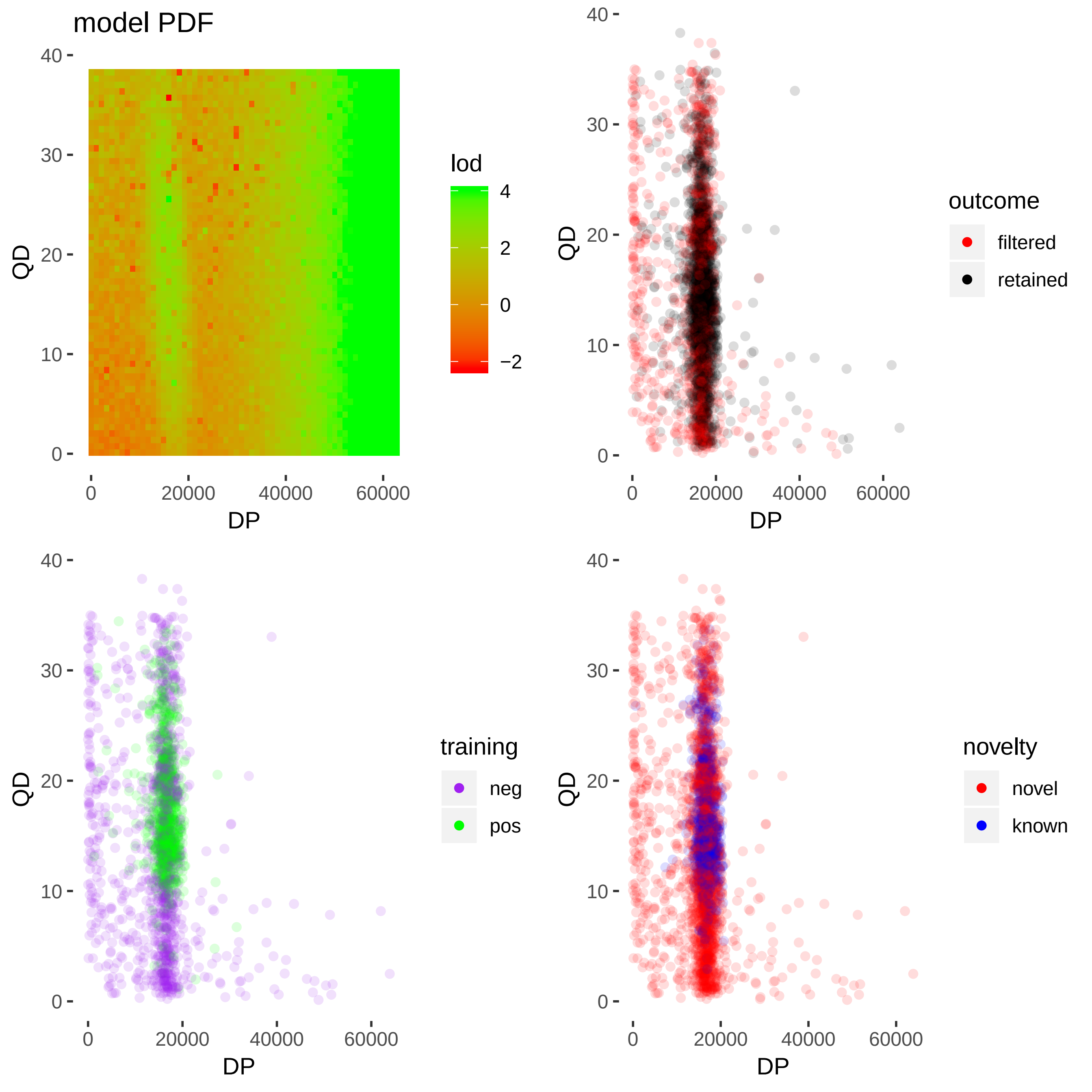

model PDF
lod
4
2
0
−2
QD
DP
0
10
20
30
40
0
20000
40000
60000
outcome
filtered
retained
training
neg
pos
novelty
novel
known

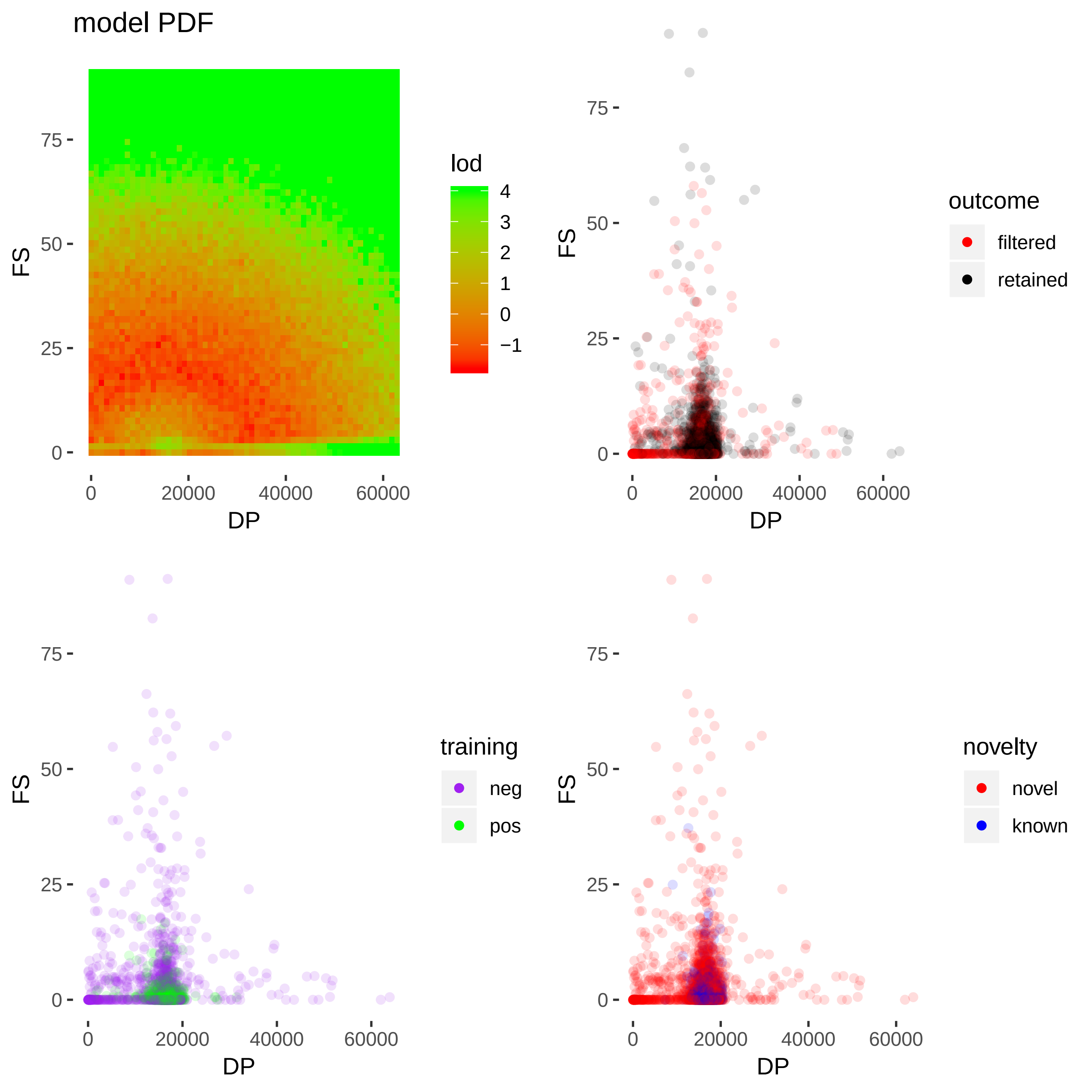
model PDF
lod
4
3
2
1
0
−1
FS
DP
0
20000
40000
60000
0
25
50
75
outcome
filtered
retained
training
neg
pos
novelty
novel
known

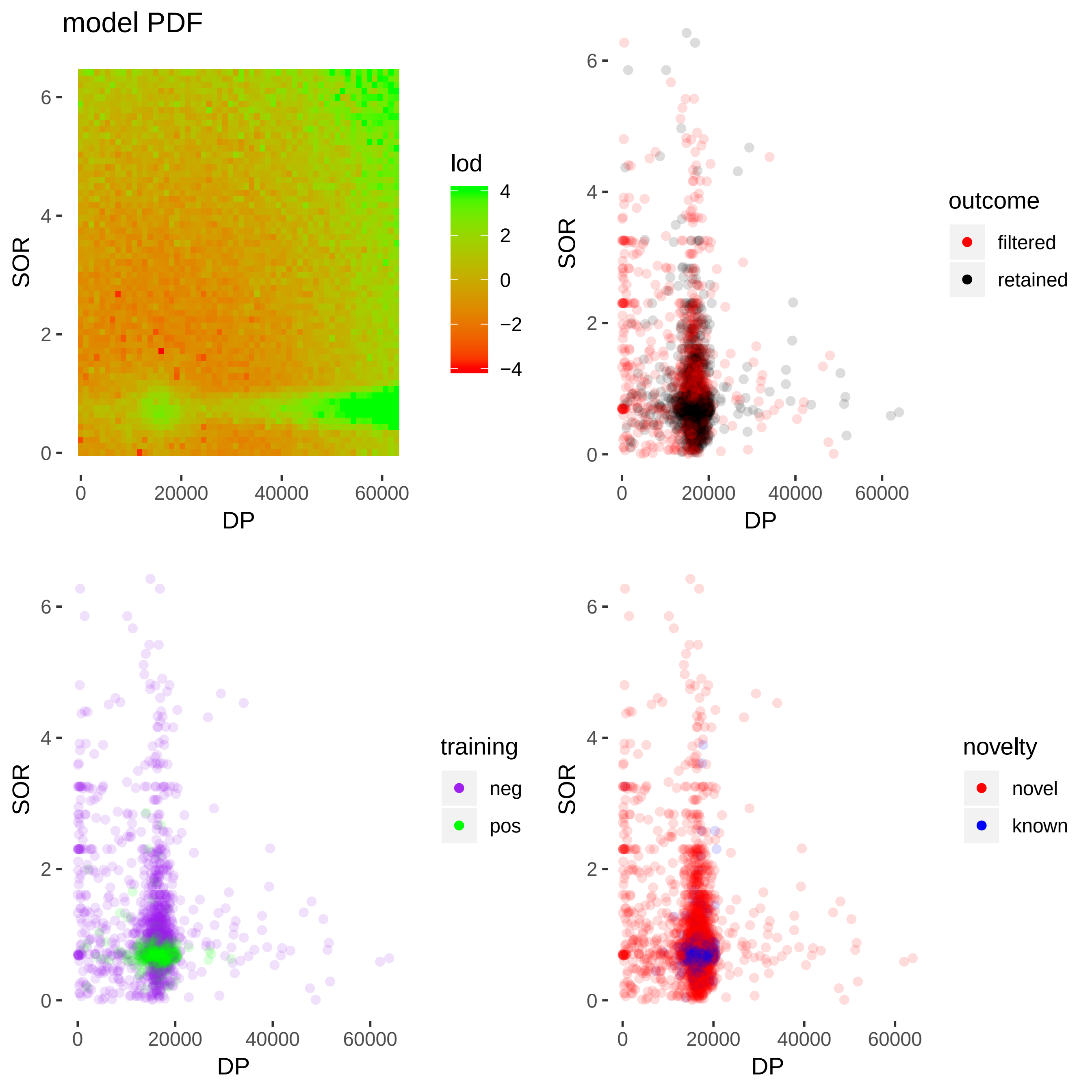

model PDF
SOR
DP
lod
4
2
0
−2
−4
outcome
filtered
retained
training
neg
pos
novelty
novel
known
0
20000
40000
60000
6

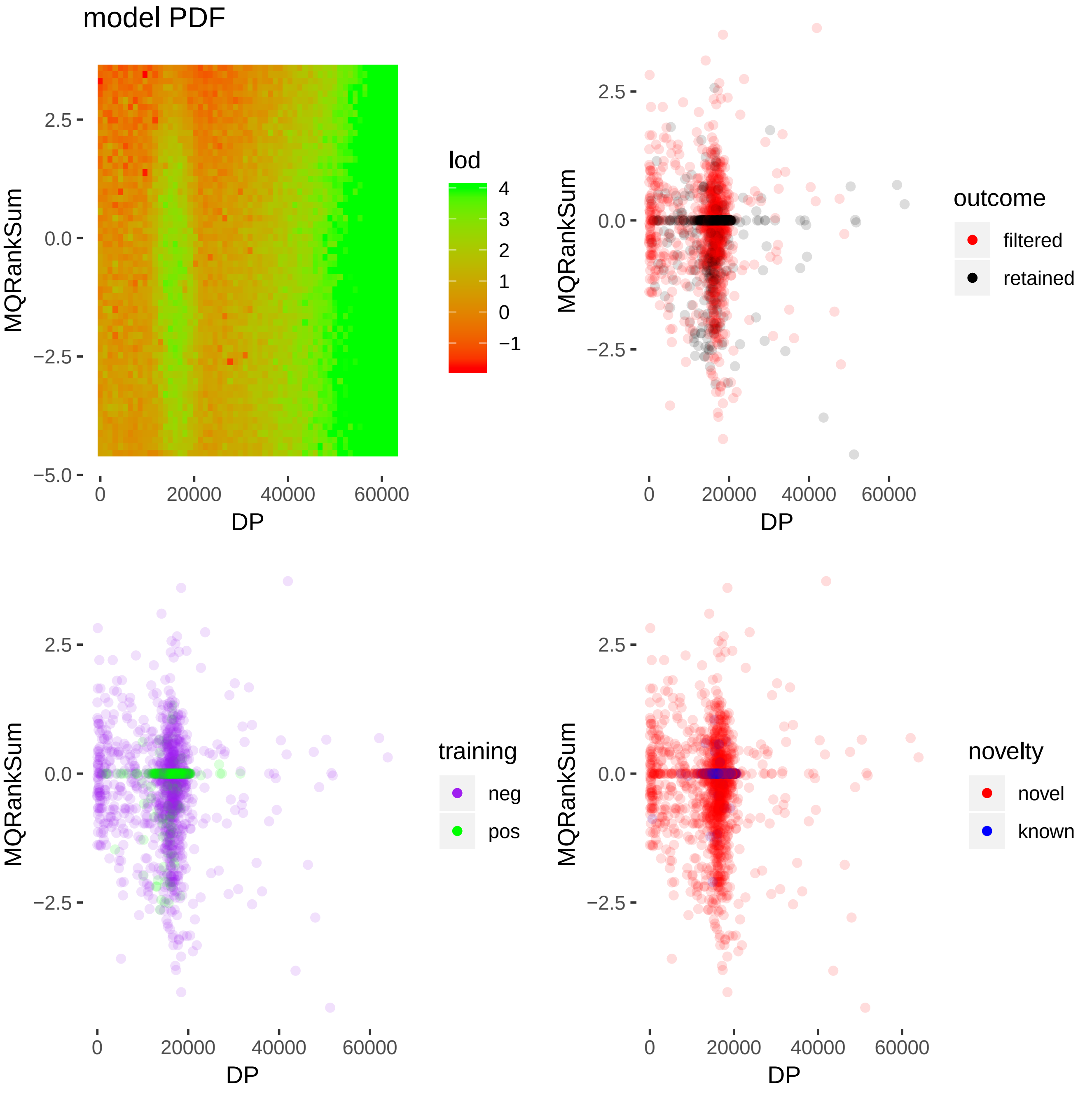
model PDF
lod
outcome
filtered
retained
training
neg
pos
novelty
novel
known
MQRankSum
DP

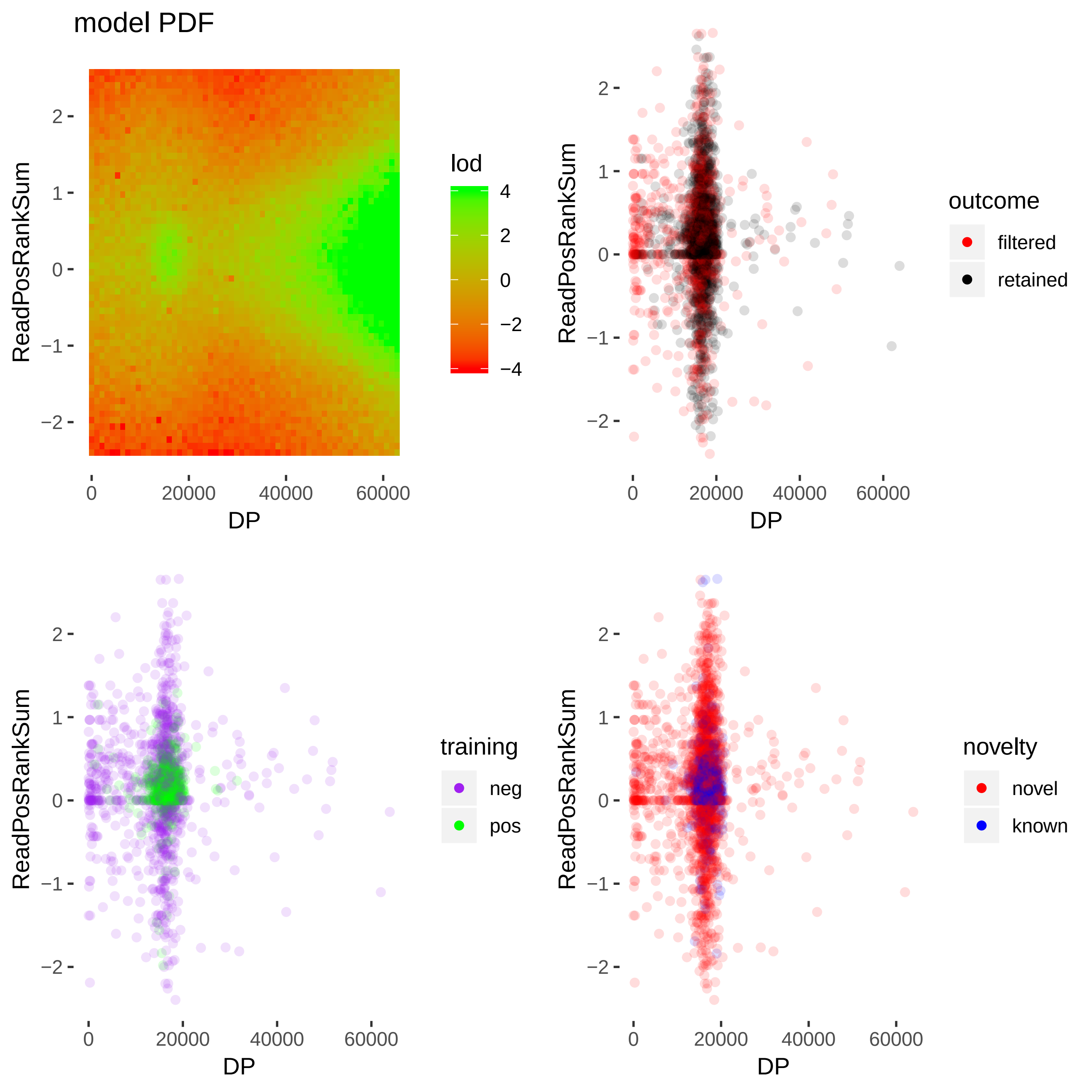

model PDF
lod
4
2
0
−2
−4
ReadPosRankSum
DP
0
20000
40000
60000
2
1
0
−1
−2
outcome
filtered
retained
training
neg
pos
novelty
novel
known

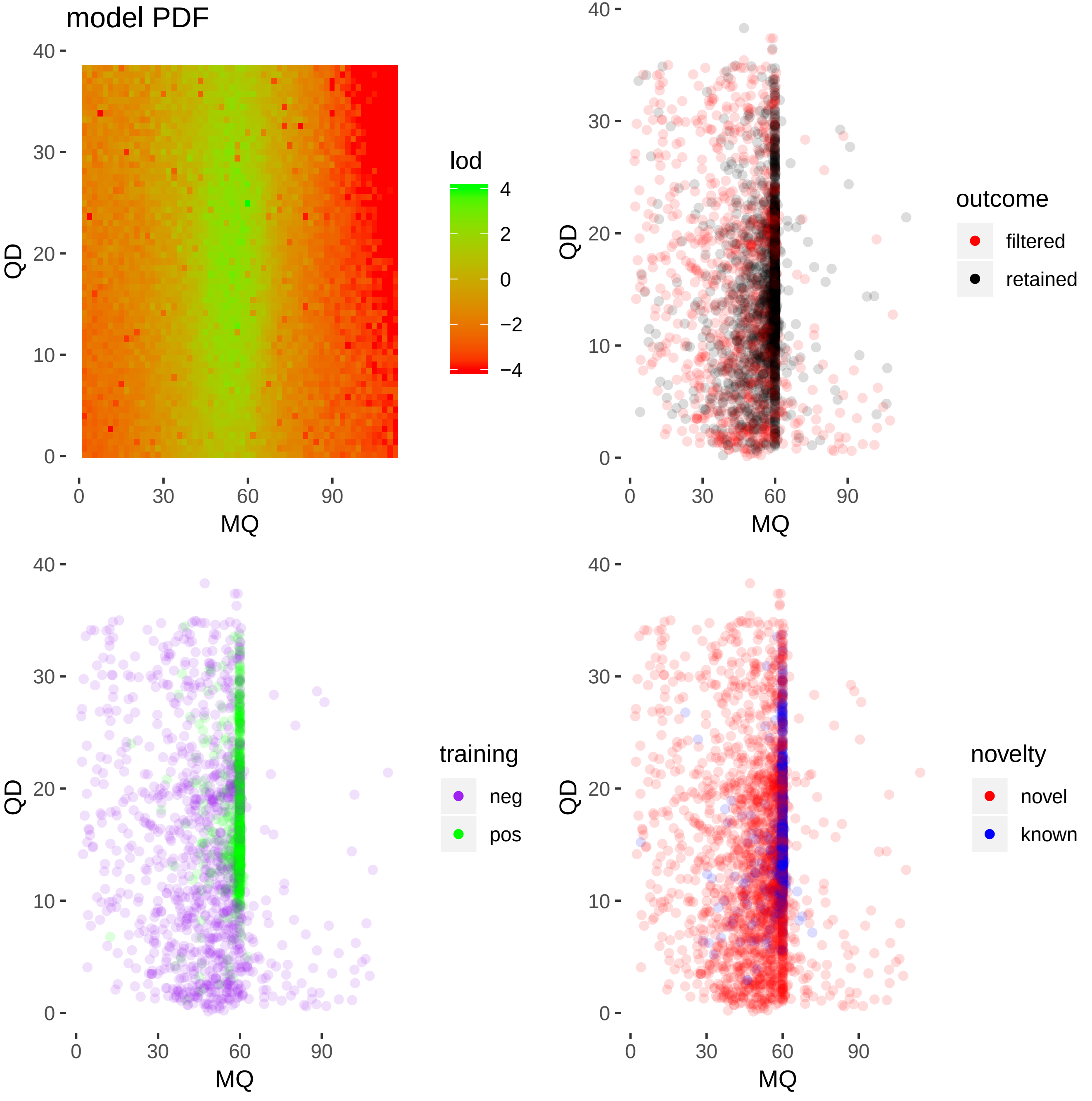

model PDF
lod
4
2
0
−2
−4
QD
MQ
outcome
filtered
retained
training
neg
pos
novelty
novel
known
0
10
20
30
40
0
30
60
90

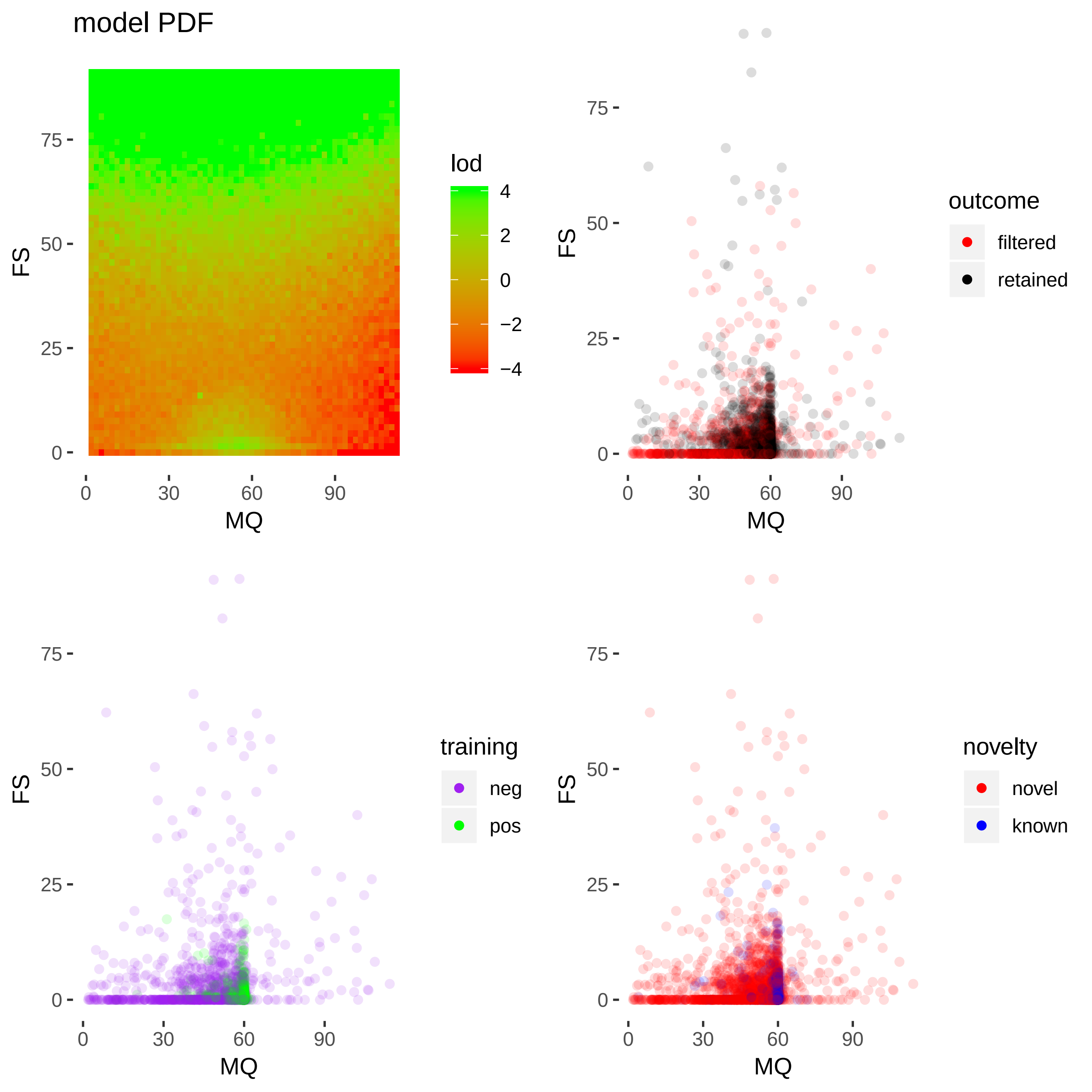

model PDF
lod
4
2
0
−2
−4
FS
MQ
outcome
filtered
retained
training
neg
pos
novelty
novel
known
75
50
25
0
30
60
90

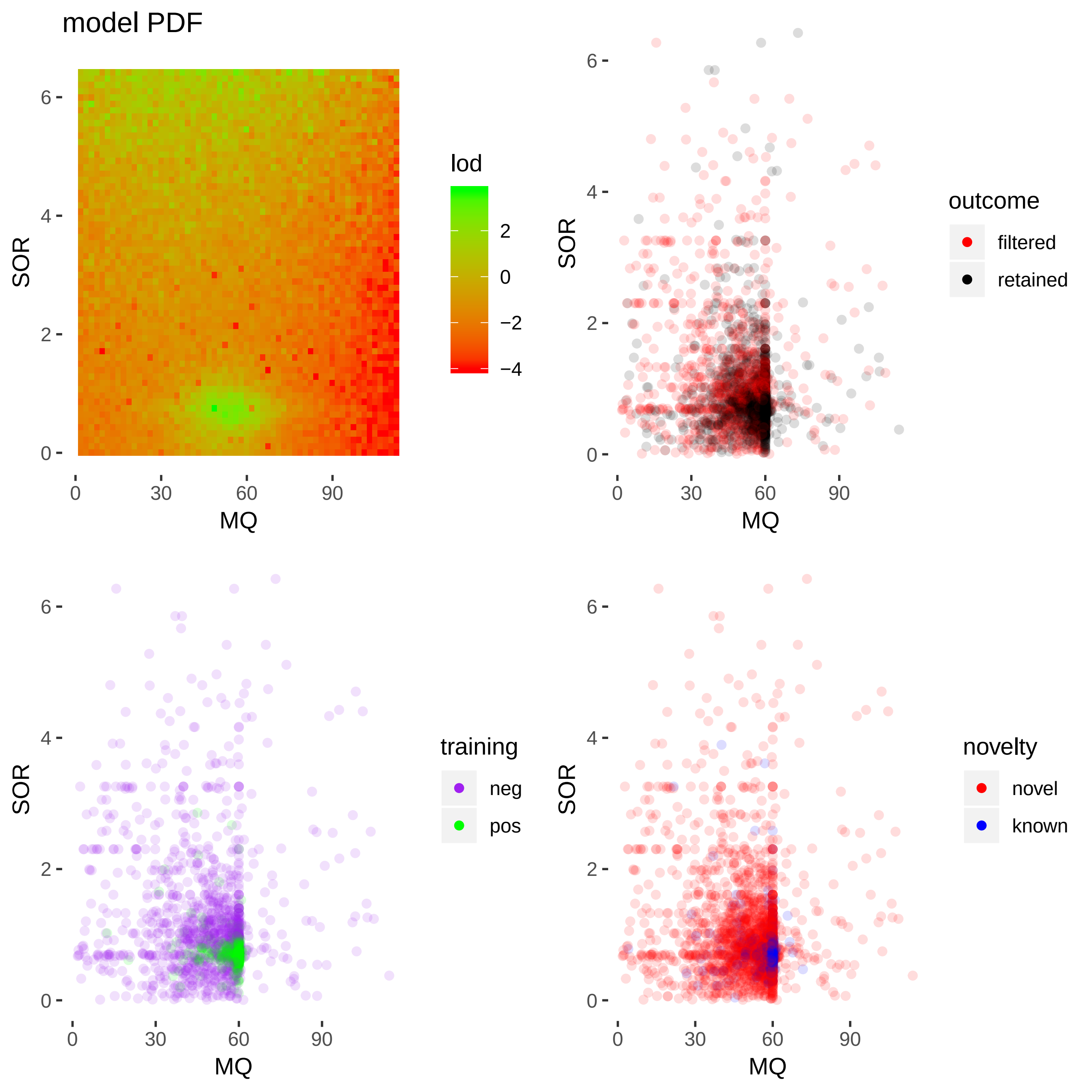
model PDF
lod
2
0
−2
−4
SOR
MQ
0
30
60
90
2
4
6
outcome
filtered
retained
training
neg
pos
novelty
novel
known

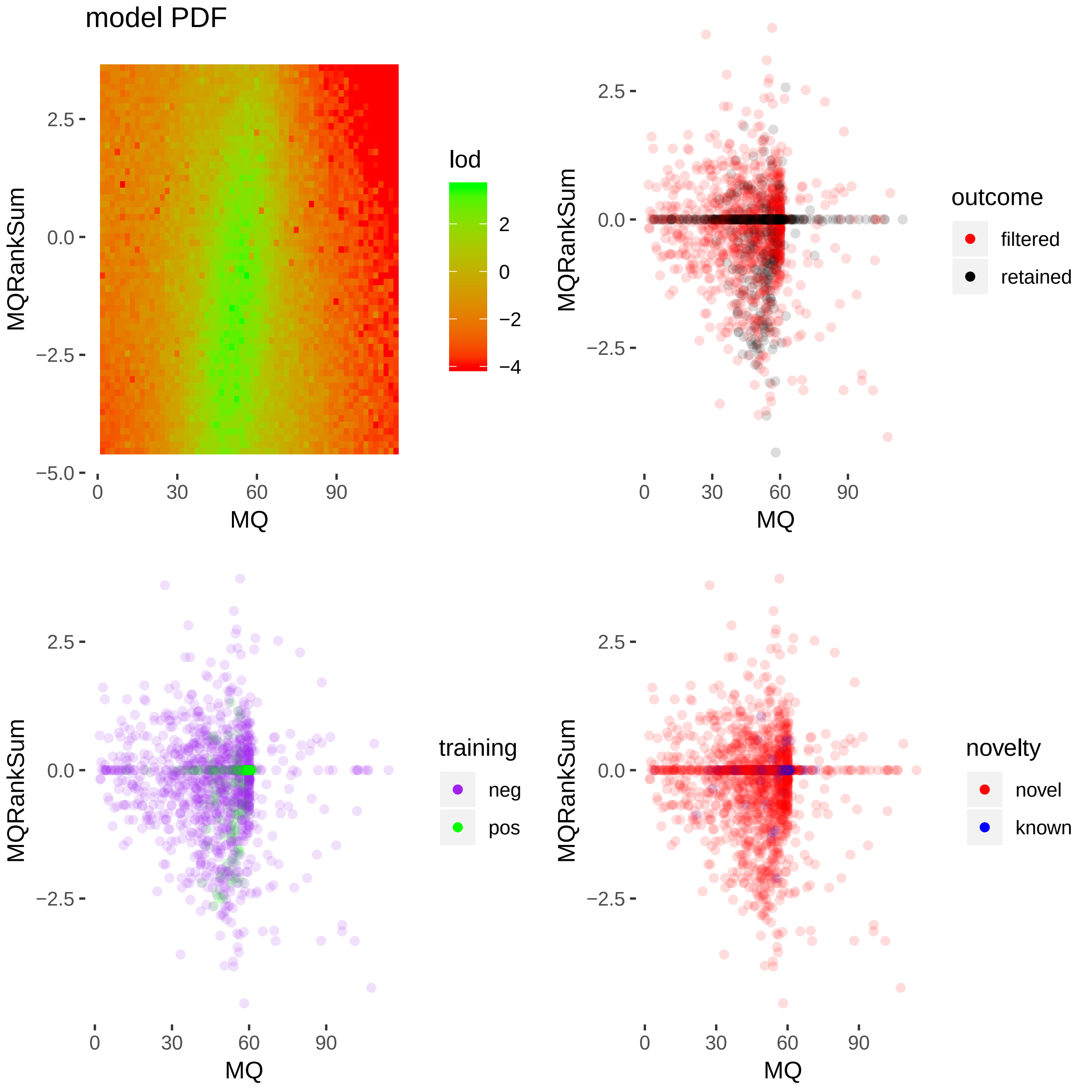
model PDF
lod
2
0
−2
−4
MQRankSum
MQ
2.5
0.0
−2.5
−5.0
0
30
60
90
outcome
filtered
retained
training
neg
pos
novelty
novel
known

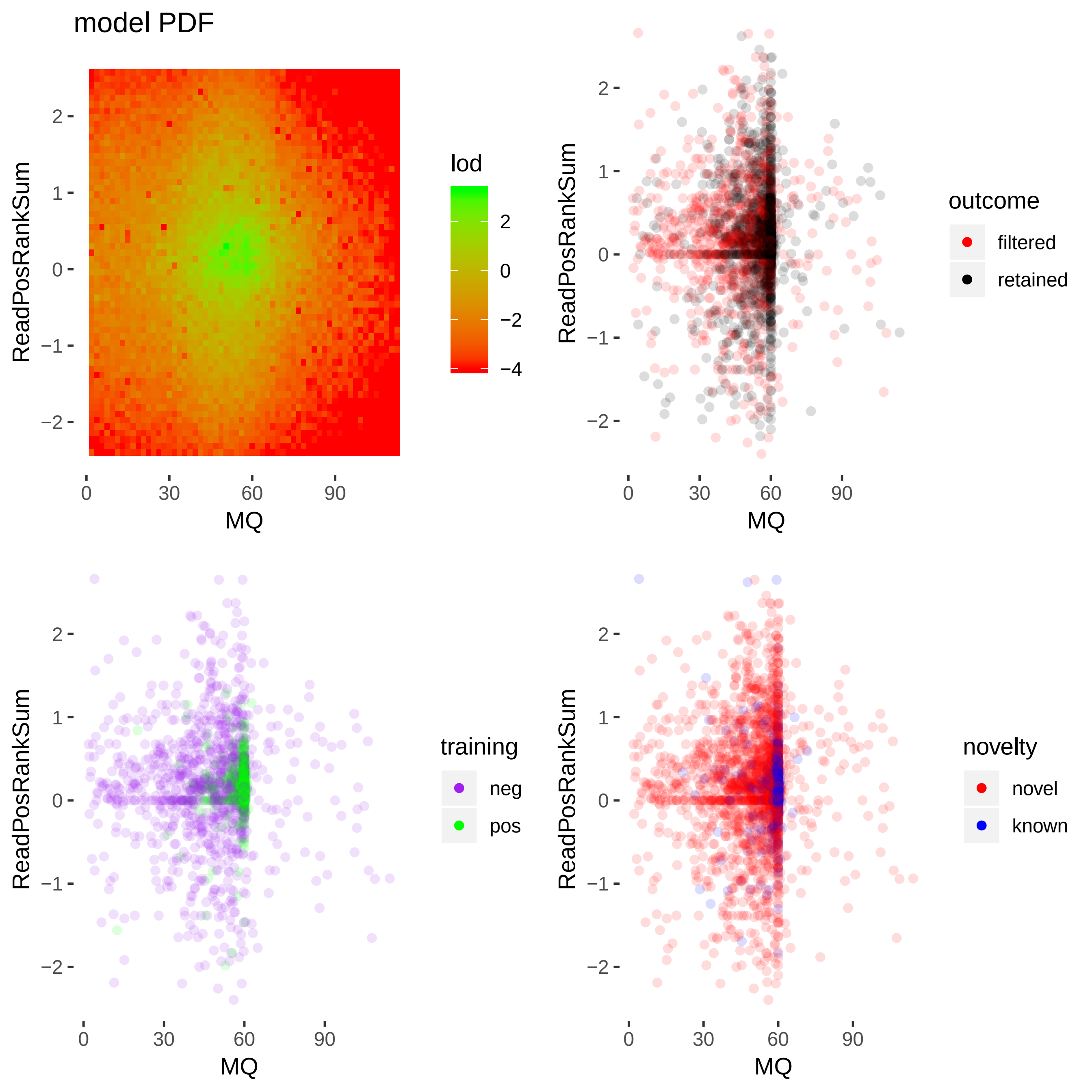

model PDF
lod
2
0
−2
−4
ReadPosRankSum
MQ
outcome
filtered
retained
training
neg
pos
novelty
novel
known

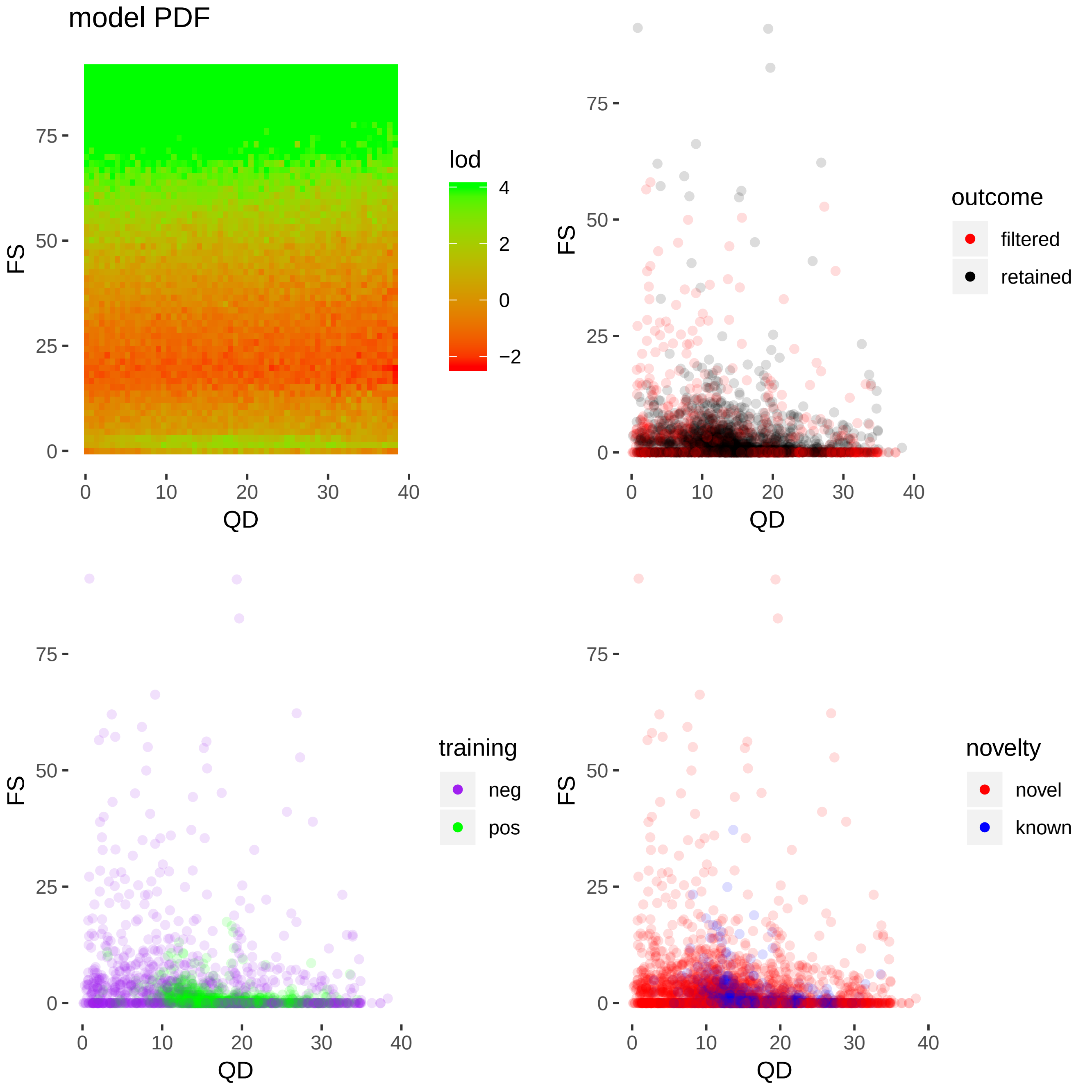

model PDF
lod
4
2
0
−2
FS
QD
0
25
50
75
0
10
20
30
40
outcome
filtered
retained
training
neg
pos
novelty
novel
known

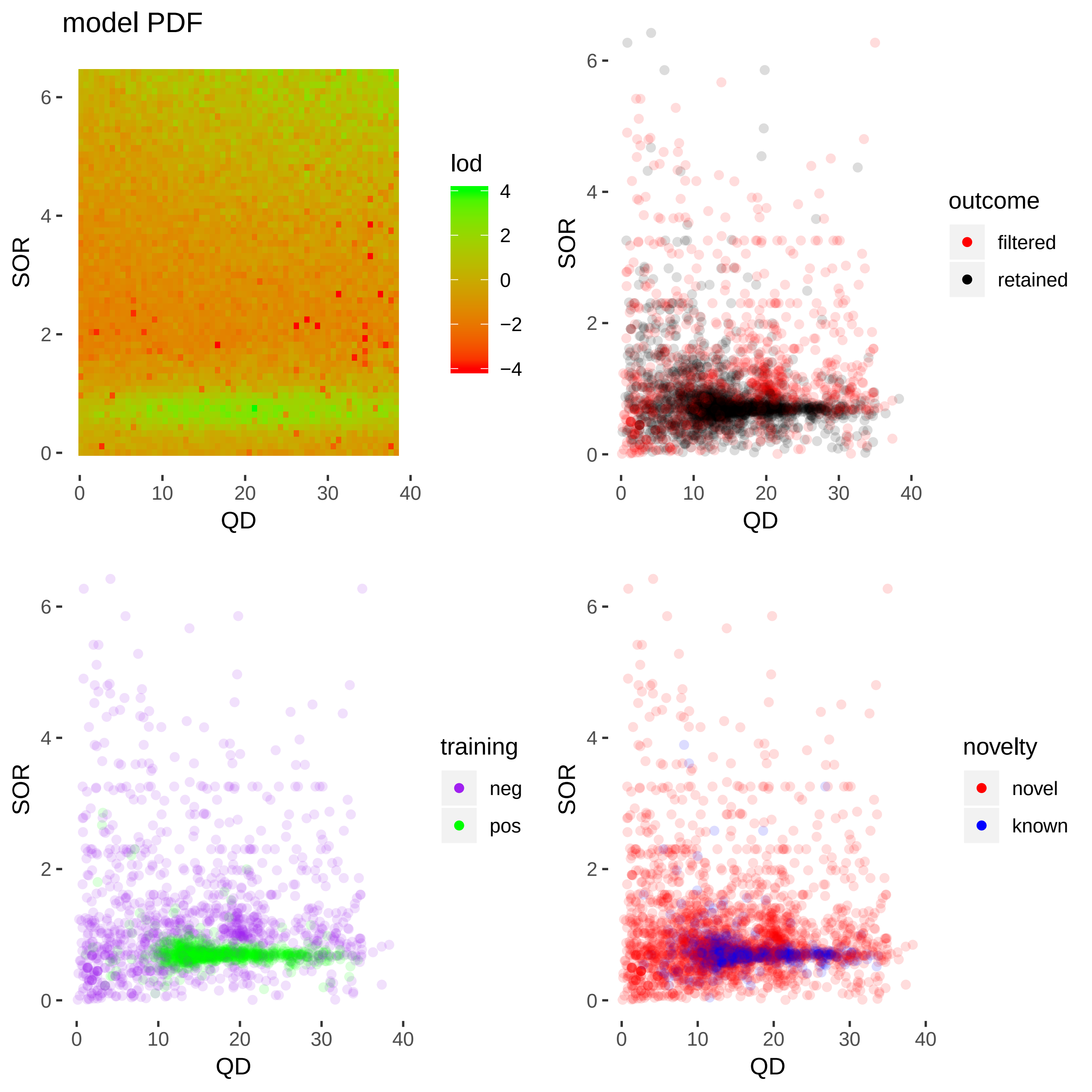

model PDF
SOR
QD
lod
4
2
0
−2
−4
outcome
filtered
retained
training
neg
pos
novelty
novel
known

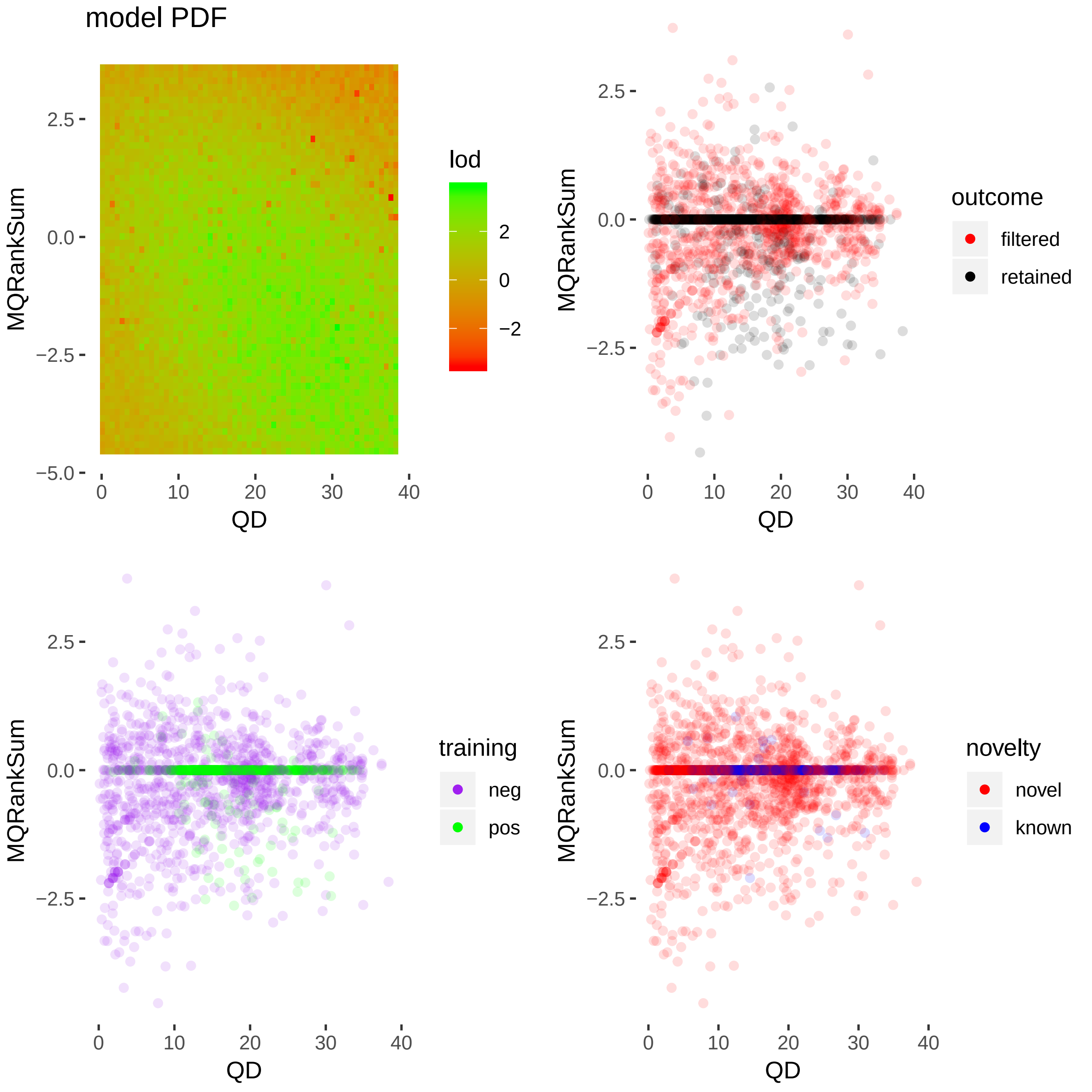

model PDF
lod
2
0
−2
outcome
filtered
retained
training
neg
pos
novelty
novel
known
MQRankSum
QD
2.5
0.0
−2.5
−5.0
0
10
20
30
40

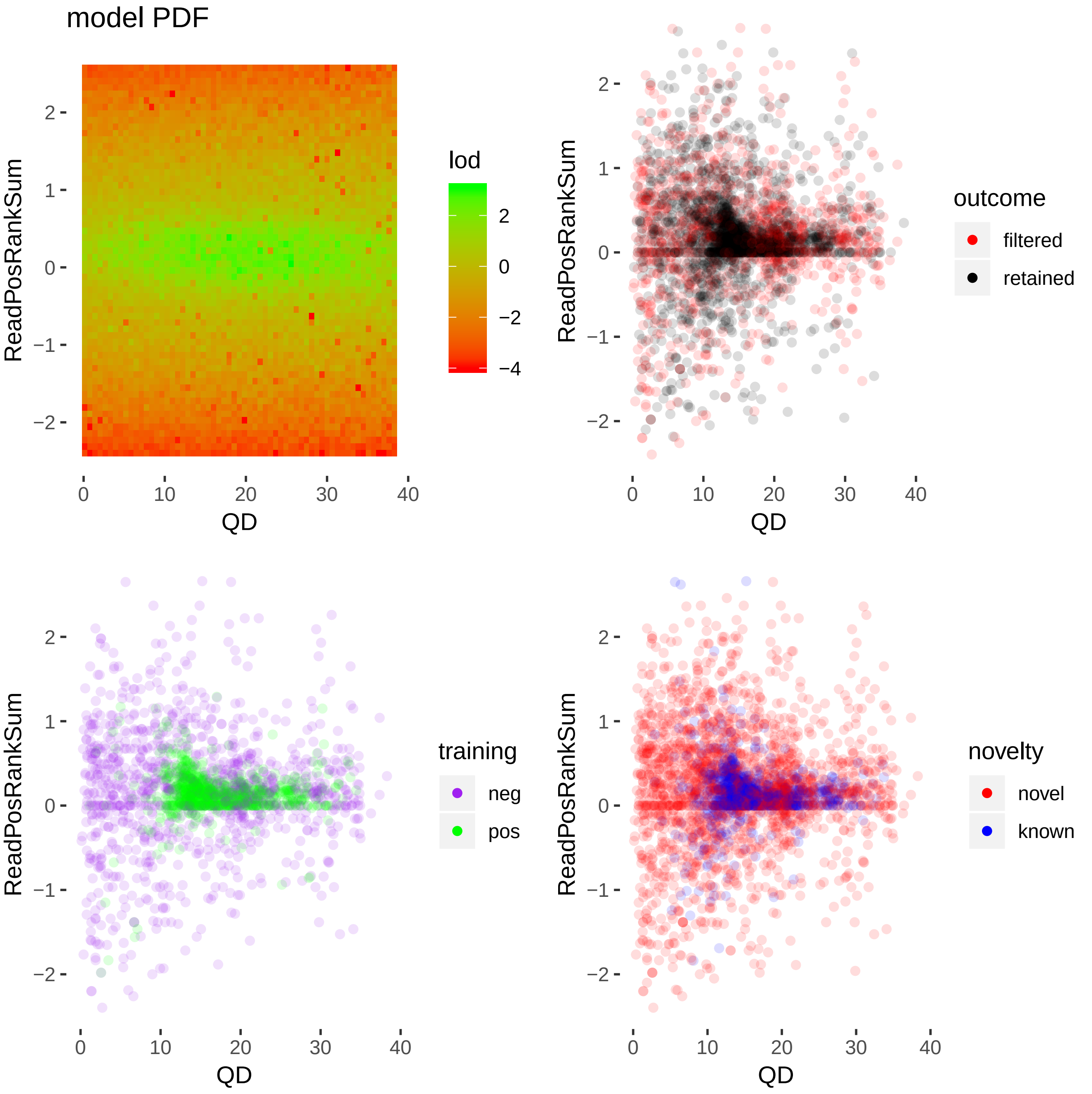

model PDF
lod
2
0
−2
−4
ReadPosRankSum
QD
outcome
filtered
retained
training
neg
pos
novelty
novel
known

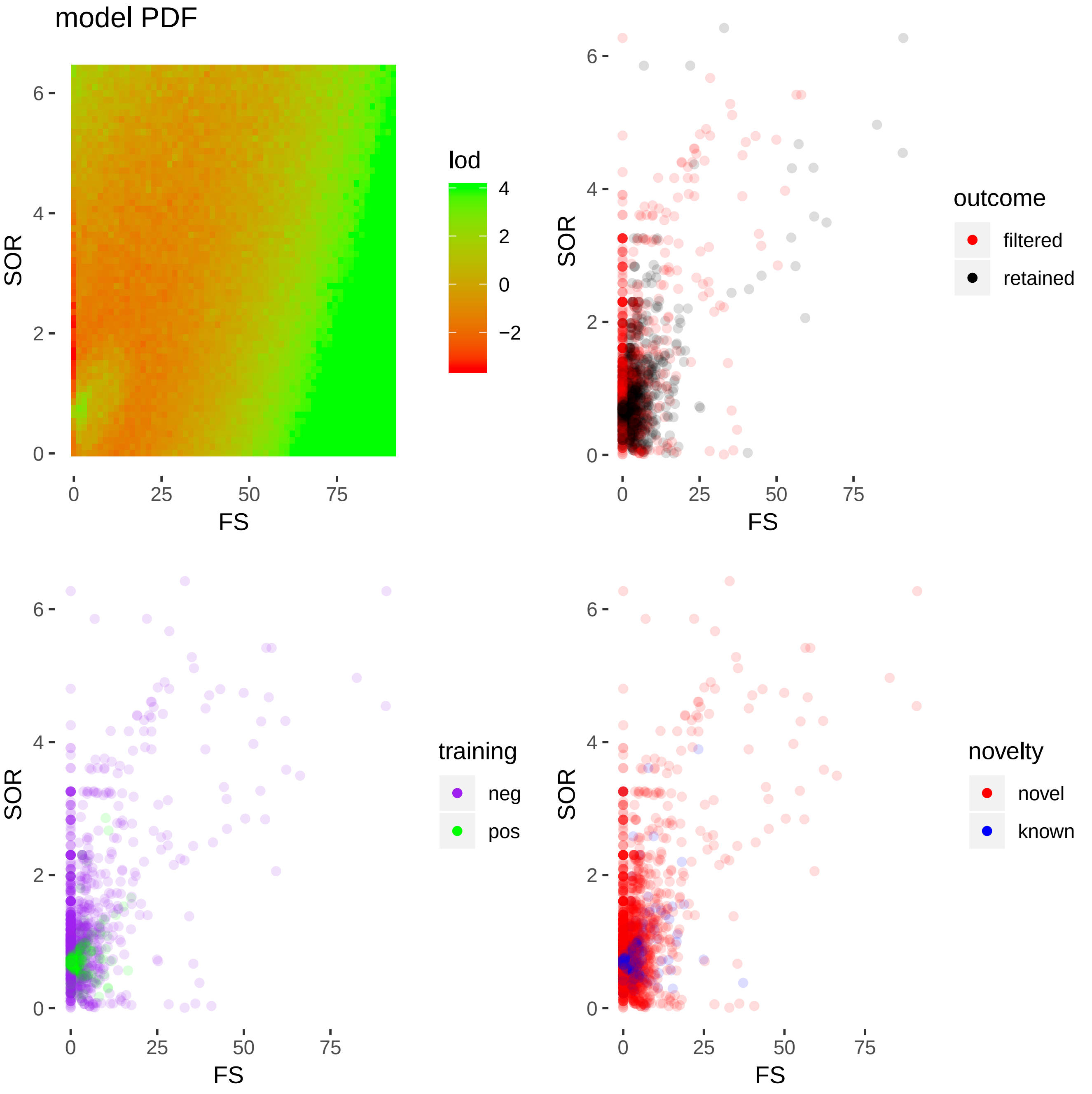
model PDF
lod
4
2
0
−2
SOR
FS
0
25
50
75
0
2
4
6
outcome
filtered
retained
training
neg
pos
novelty
novel
known

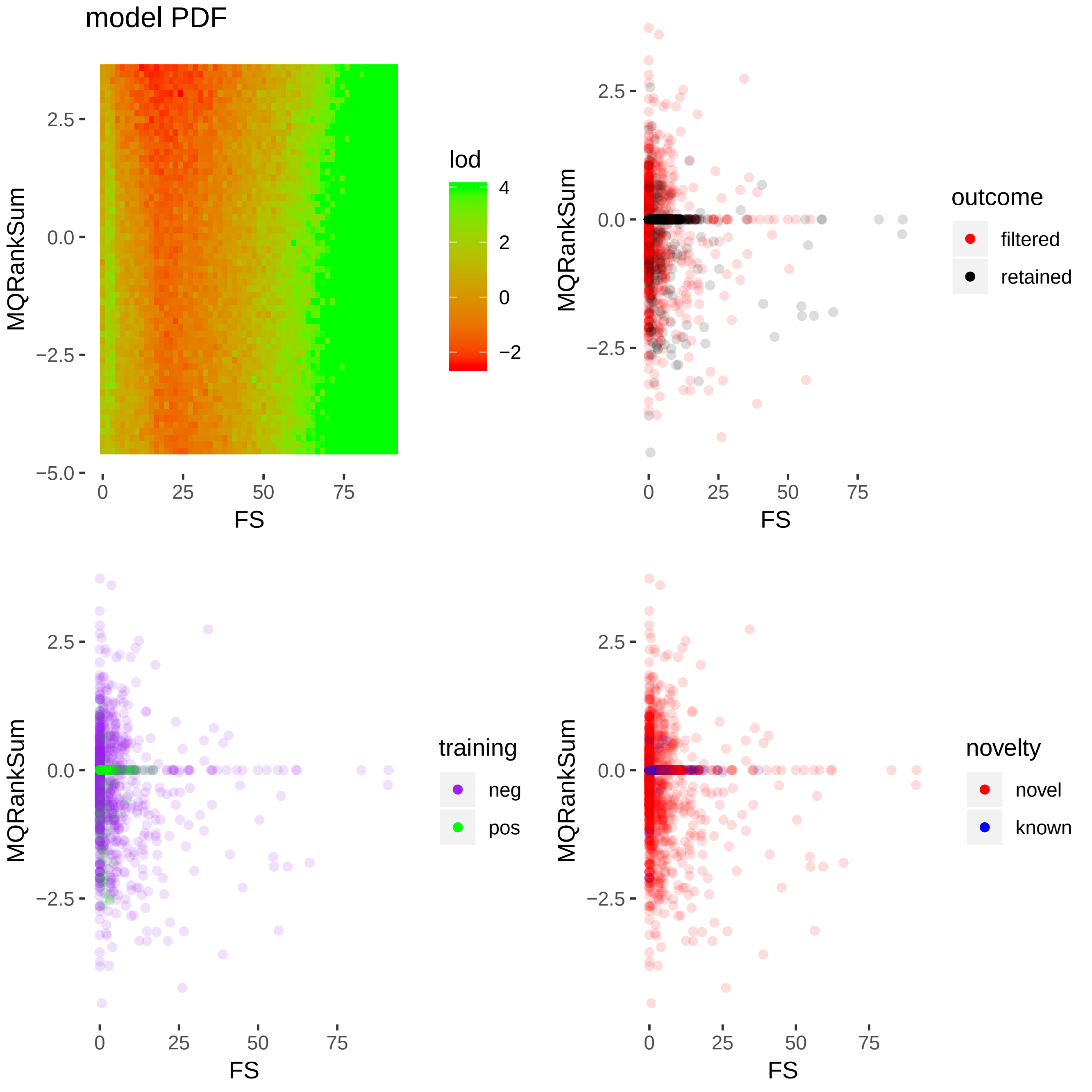

model PDF
lod
4
2
0
−2
MQRankSum
FS
outcome
filtered
retained
training
neg
pos
novelty
novel
known

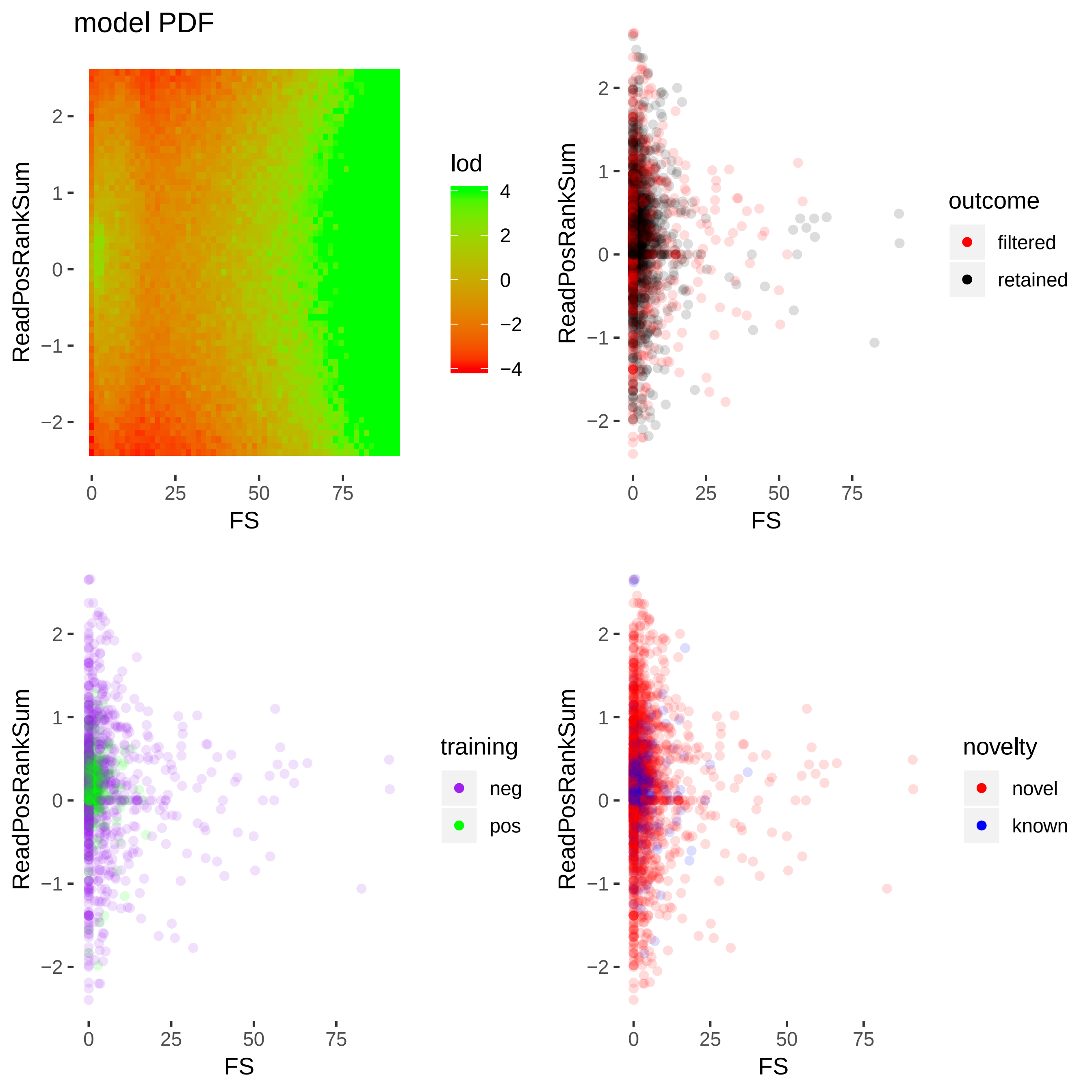

model PDF
ReadPosRankSum
FS
lod
4
2
0
−2
−4
2
1
0
−1
−2
0
25
50
75
outcome
filtered
retained
training
neg
pos
novelty
novel
known

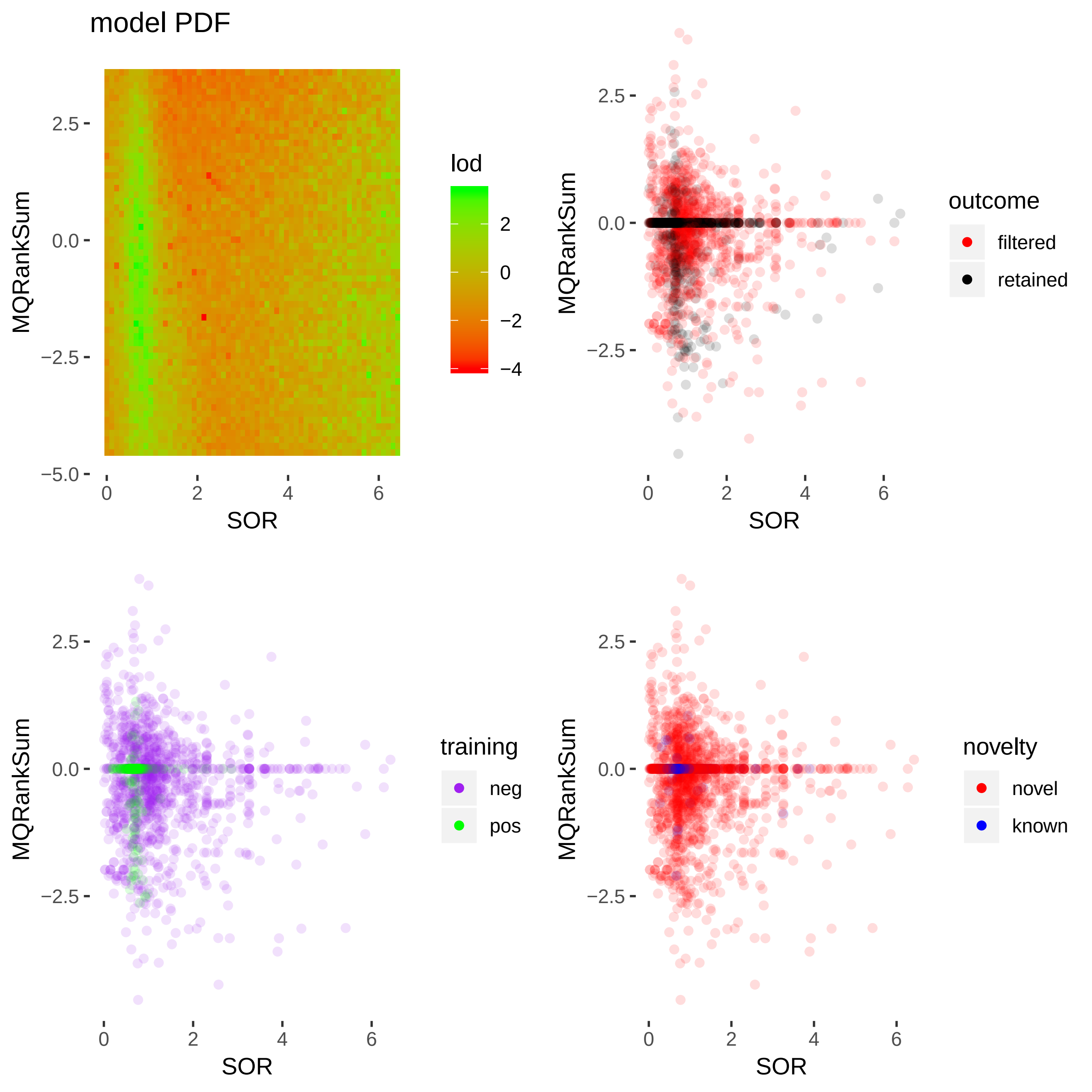

model PDF
lod
2
0
−2
−4
MQRankSum
SOR
2.5
0.0
−2.5
−5.0
0
2
4
6
outcome
filtered
retained
training
neg
pos
novelty
novel
known

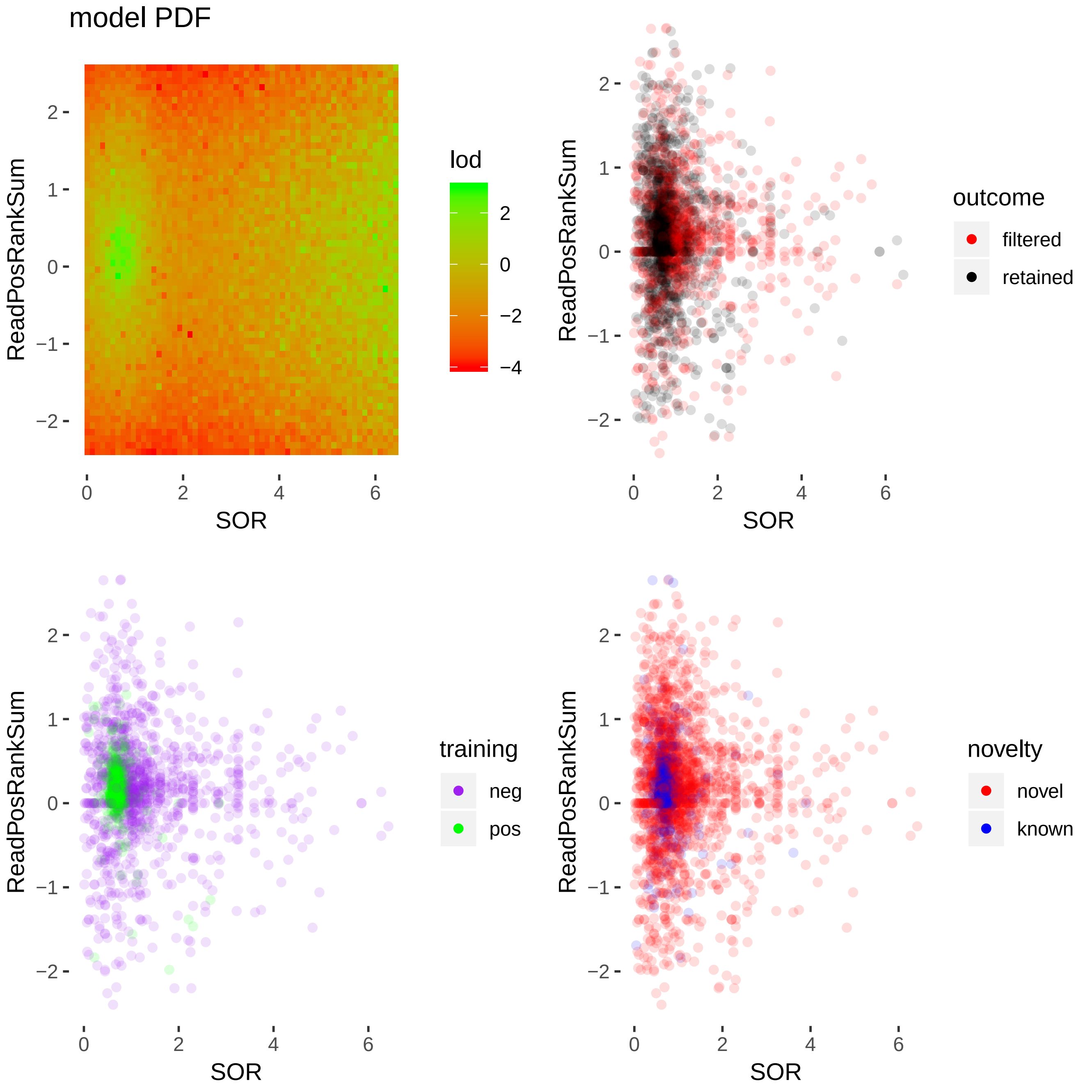

model PDF
ReadPosRankSum
SOR
lod
outcome
filtered
retained
training
neg
pos
novelty
novel
known

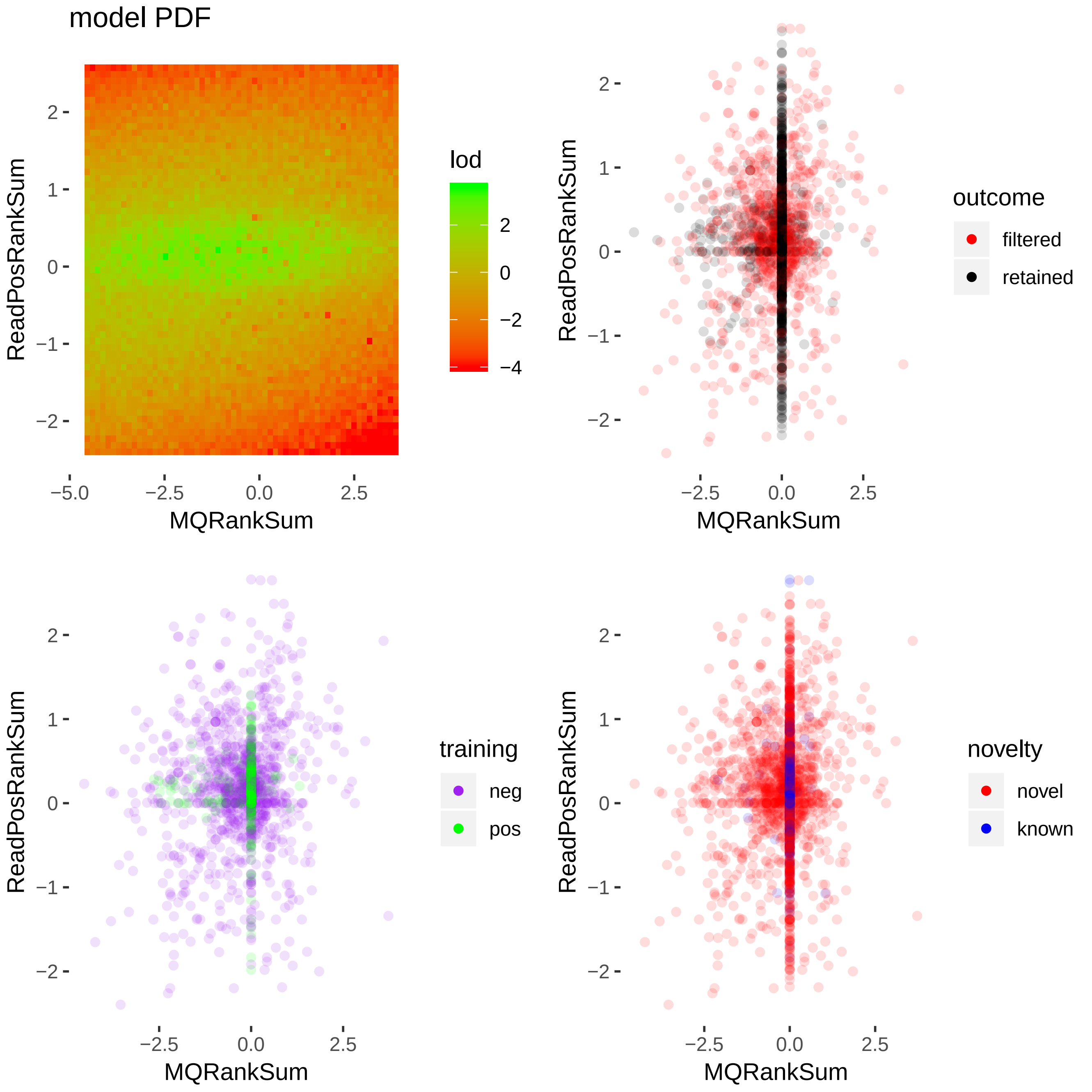

model PDF
lod
2
0
−2
−4
ReadPosRankSum
MQRankSum
−5.0
−2.5
0.0
2.5
outcome
filtered
retained
training
neg
pos
novelty
novel
known
