# Supplementary material for: VarGoats project: a dataset of 1159 whole-genome sequences to dissect Capra hircus global diversity
Source: Genet Sel Evol. 2021 Nov 8;53:86. doi: 10.1186/s12711-021-00659-6 (PMC8573910; doi:10.1186/s12711-021-00659-6)
Supplement: Supplementary file 5 — Additional file 5: Figure S3. Pairwise comparisons of the annotations chosen in VQSR for InDels. Description: Modeling report generated by GATK VariantRecalibrator for every pairwise combination of annotations used (QD, DP, FS, MQRankSum, ReadPosRankSum and SOR), with a 2D projection of the Gaussian mixture model. [file 12711_2021_659_MOESM5_ESM.pdf]

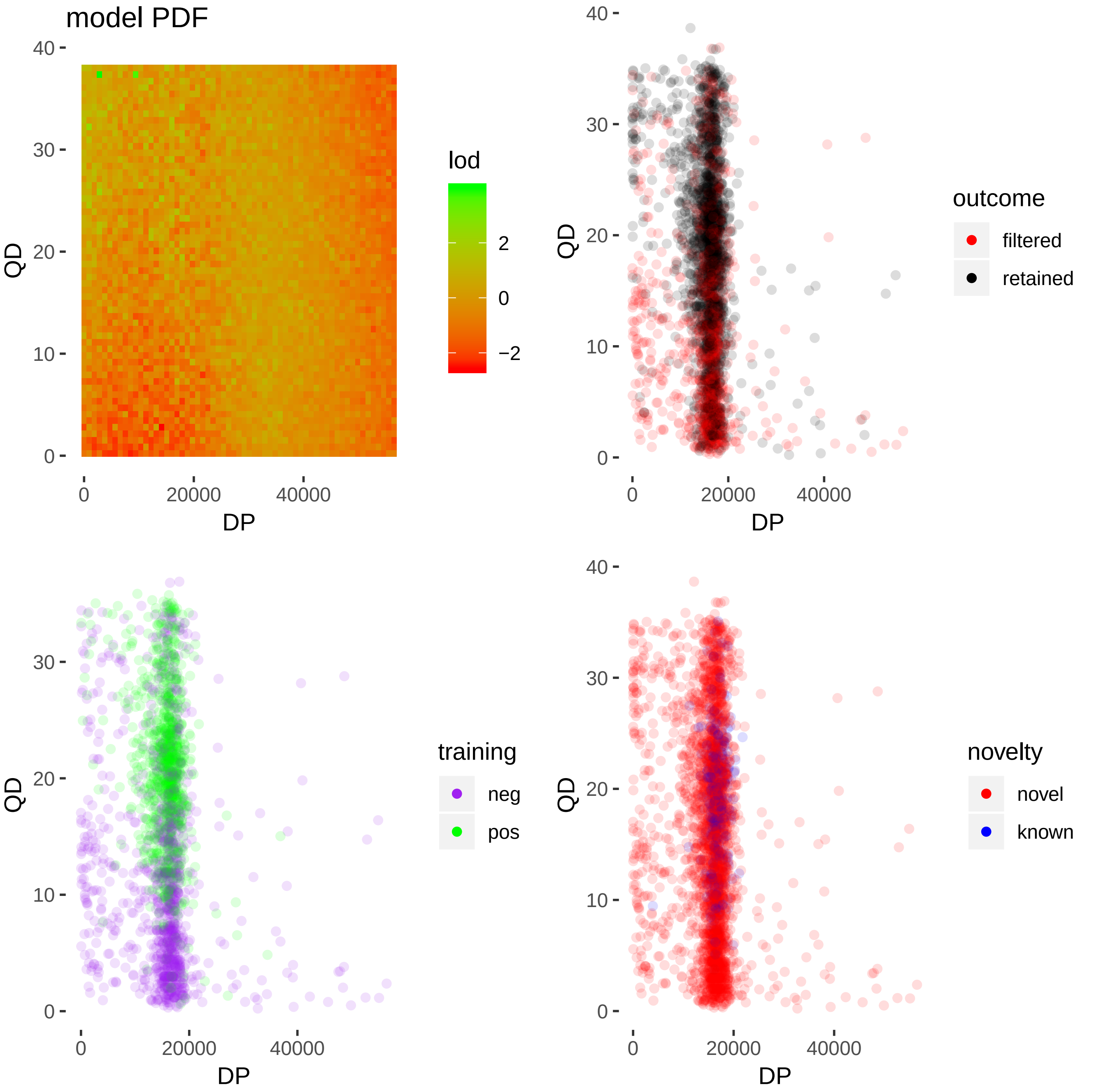

model PDF
QD
DP
lod
2
0
−2
0
10
20
30
40
20000
40000
outcome
filtered
retained
training
neg
pos
novelty
novel
known

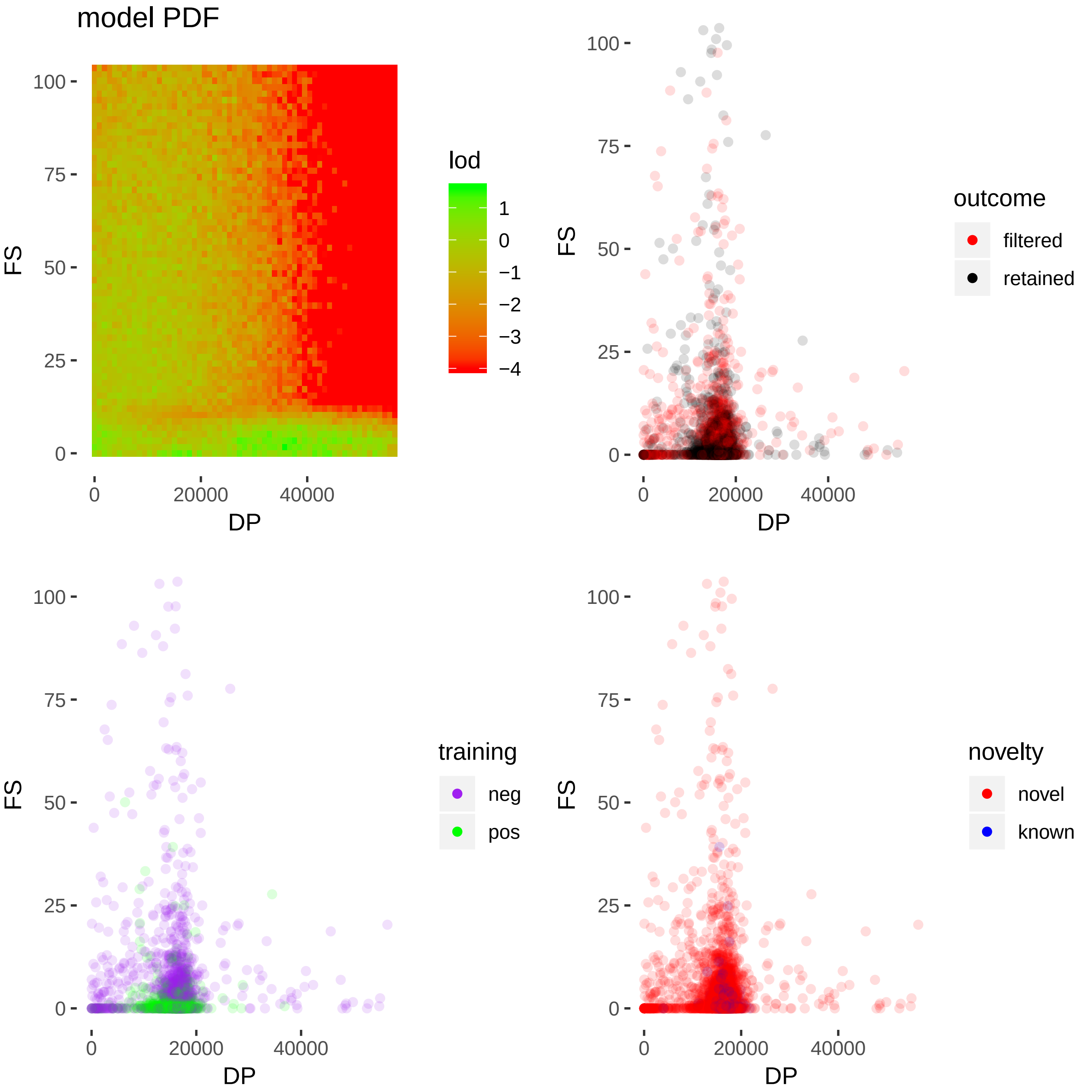

model PDF
lod
1
0
−1
−2
−3
−4
FS
DP
100
75
50
25
0
20000
40000
outcome
filtered
retained
training
neg
pos
novelty
novel
known

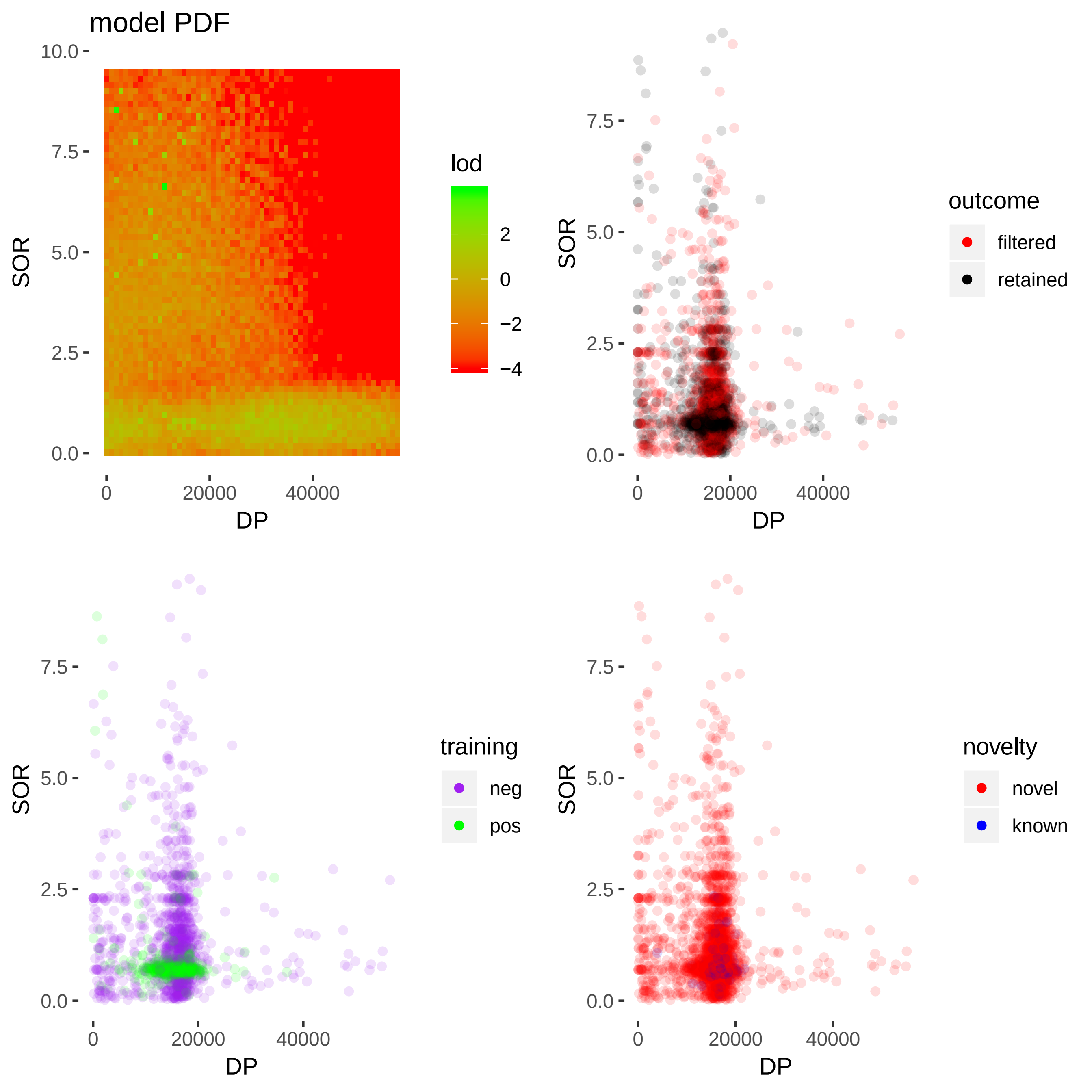

model PDF
lod
2
0
−2
−4
SOR
DP
10.0
7.5
5.0
2.5
0.0
0
20000
40000
outcome
filtered
retained
training
neg
pos
novelty
novel
known

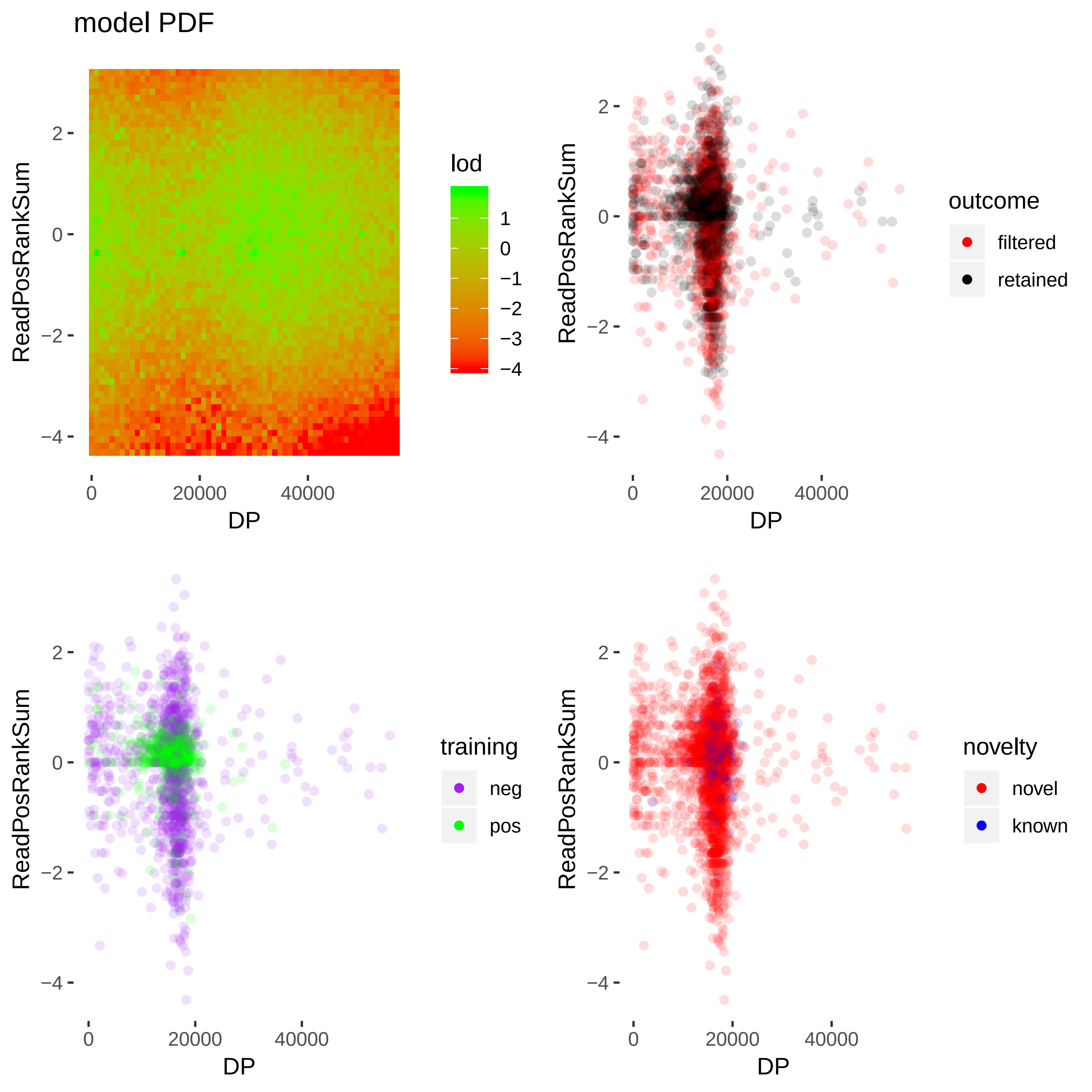
model PDF
ReadPosRankSum
DP
lod
outcome
filtered
retained
training
neg
pos
novelty
novel
known

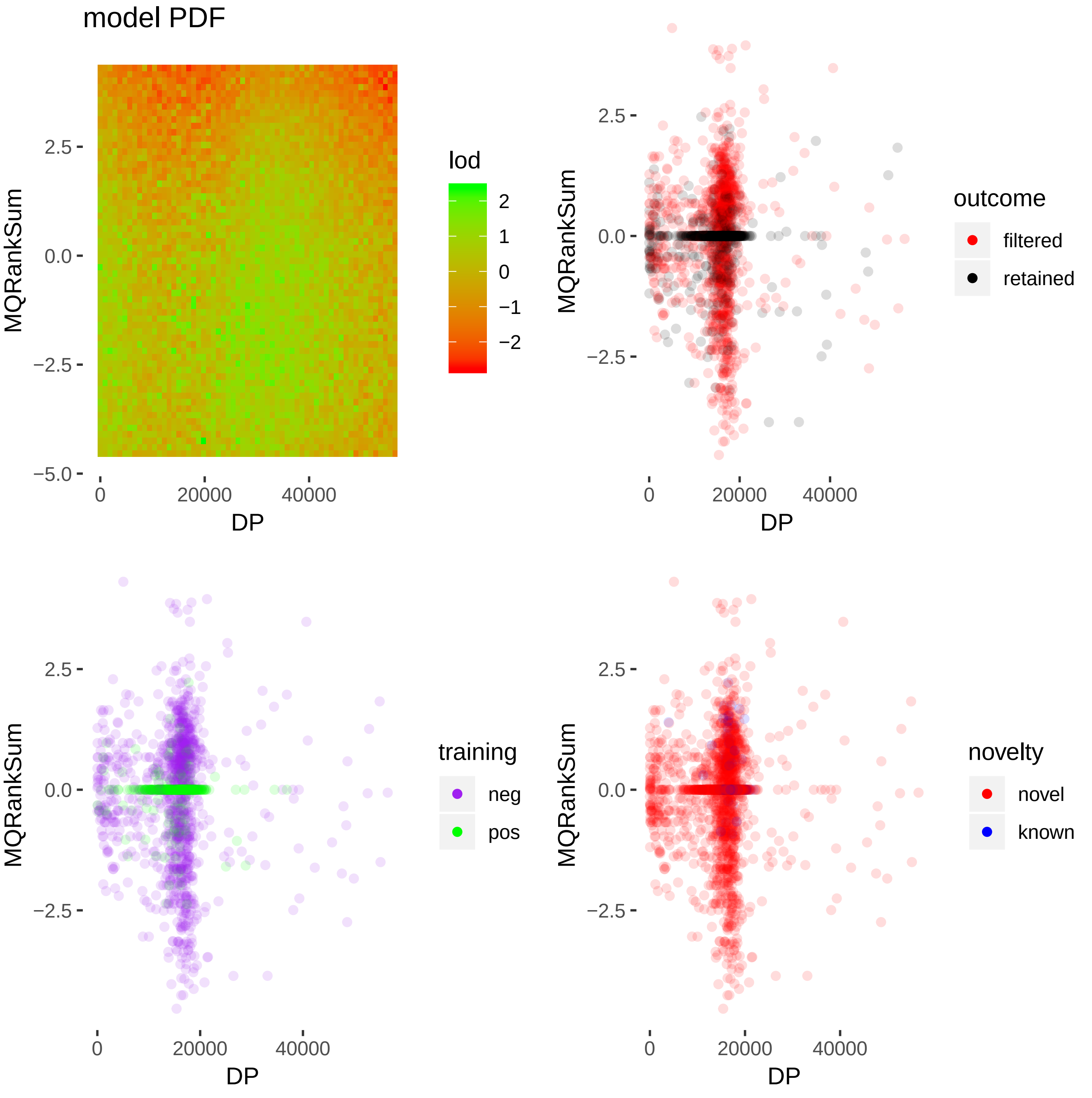

model PDF
MQRankSum
DP
lod
2
1
0
−1
−2
2.5
0.0
−2.5
−5.0
0
20000
40000
outcome
filtered
retained
training
neg
pos
novelty
novel
known

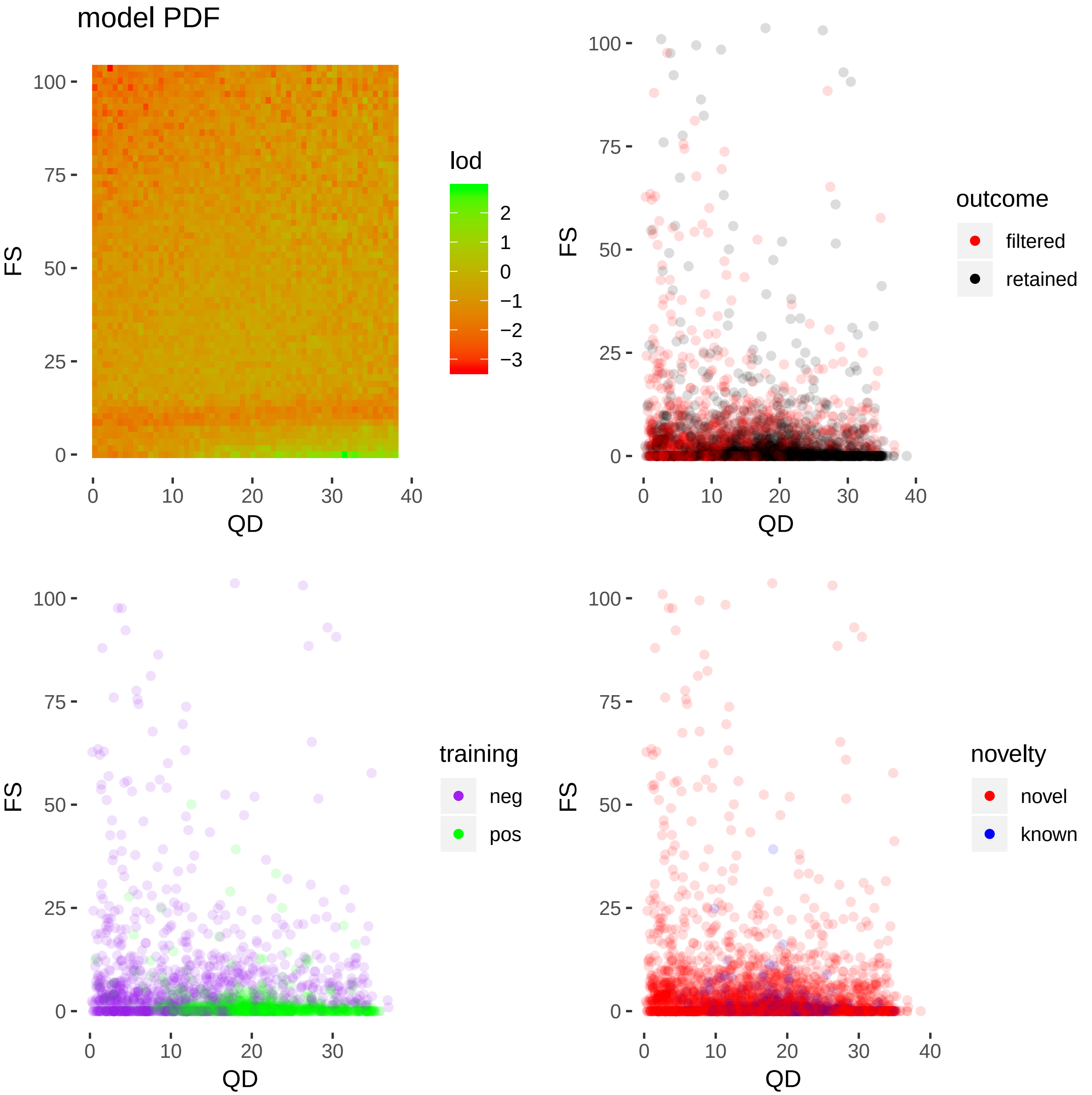

model PDF
FS
QD
lod
2
1
0
−1
−2
−3
outcome
filtered
retained
training
neg
pos
novelty
novel
known
0
25
50
75
100
10
20
30
40

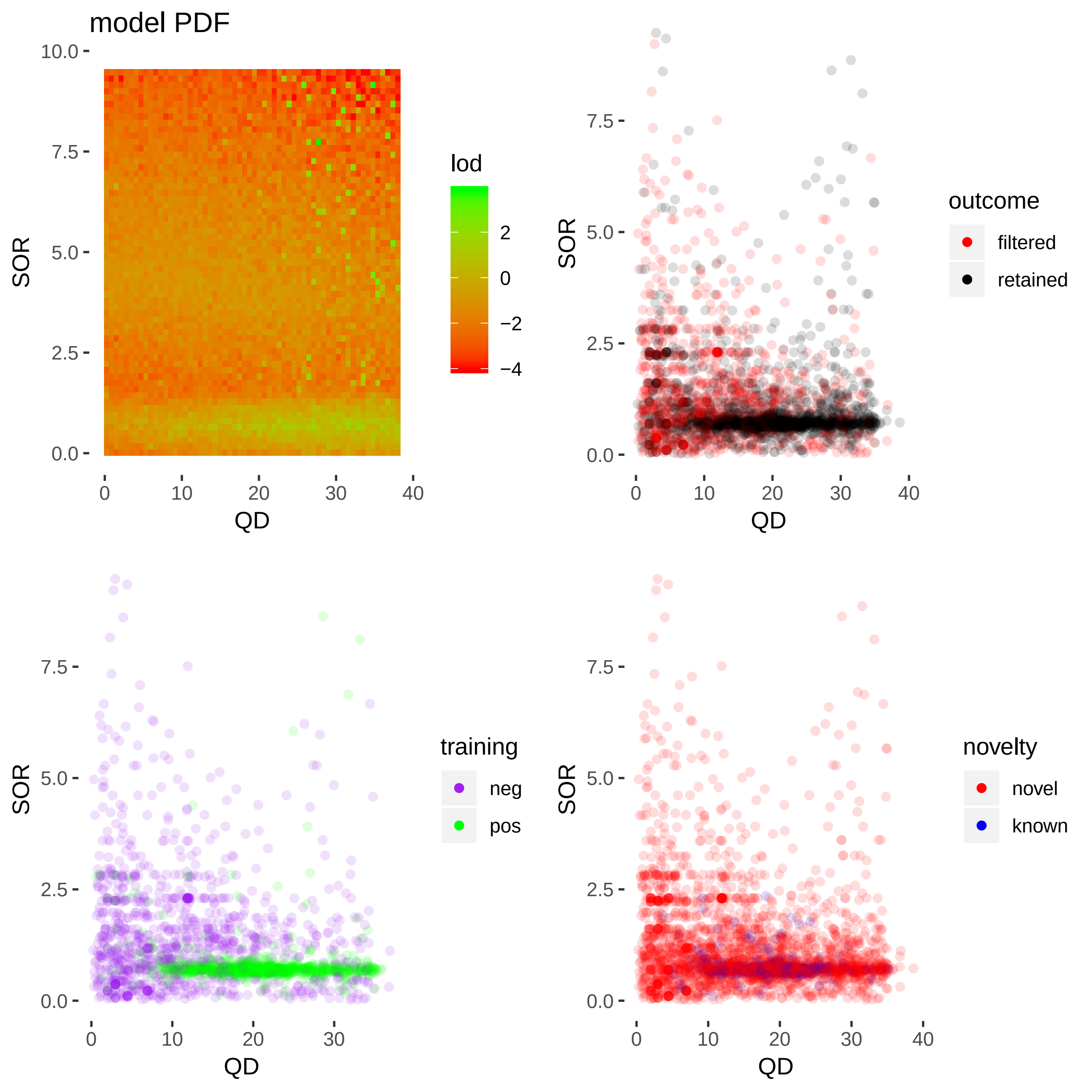

model PDF
lod
outcome
filtered
retained
training
neg
pos
novelty
novel
known
SOR
QD

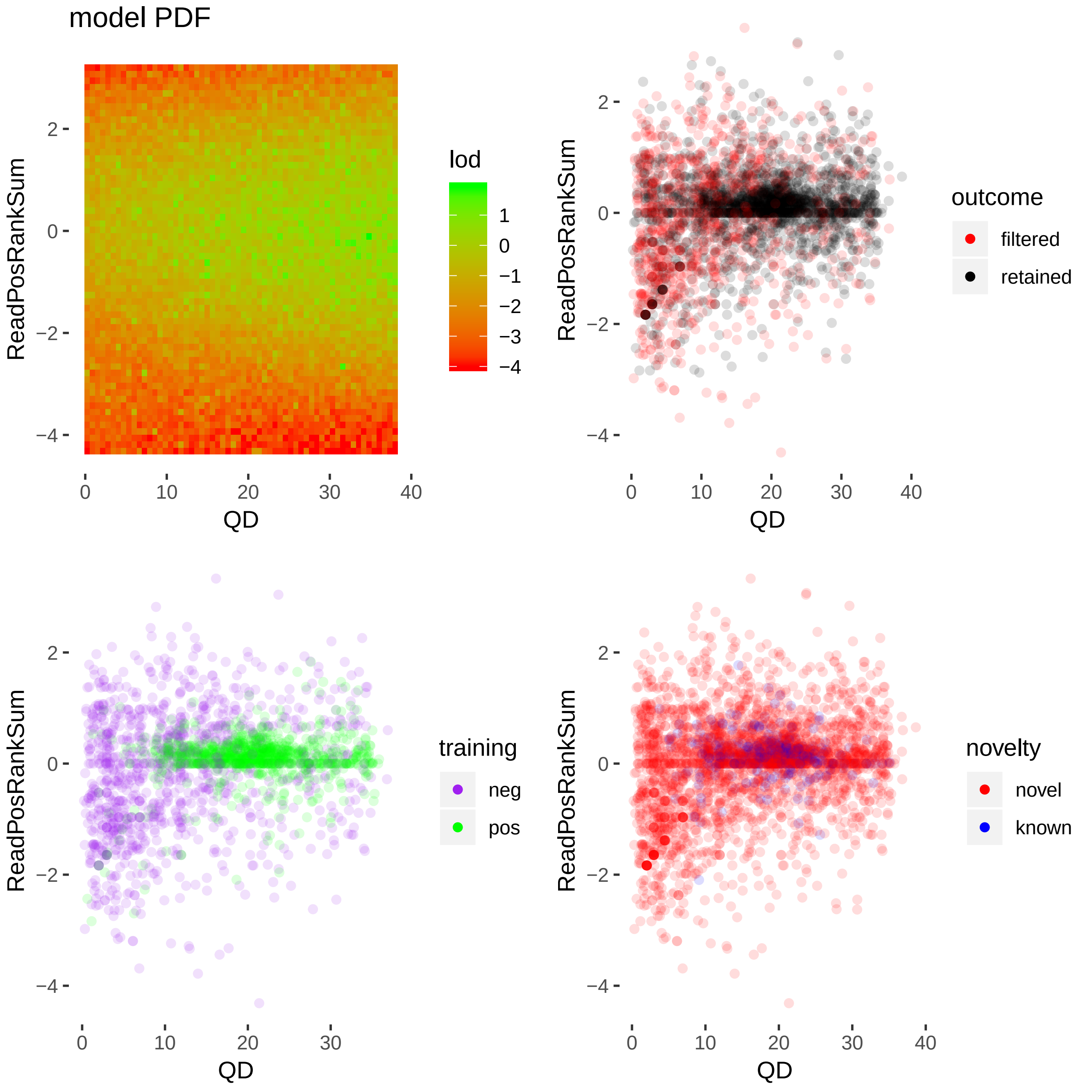
model PDF
lod
1
0
−1
−2
−3
−4
ReadPosRankSum
QD
outcome
filtered
retained
training
neg
pos
novelty
novel
known

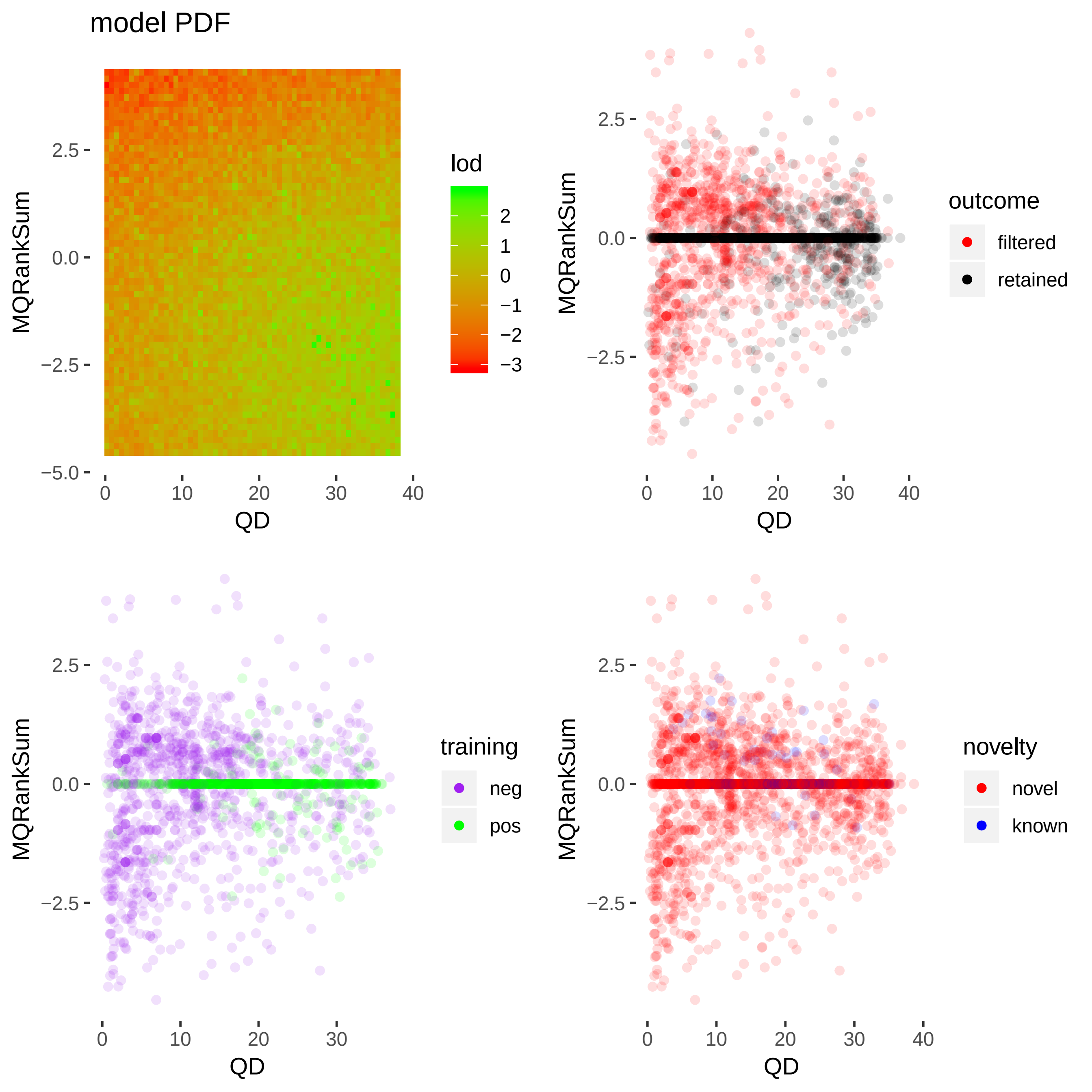
model PDF
MQRankSum
QD
lod
outcome
filtered
retained
training
neg
pos
novelty
novel
known

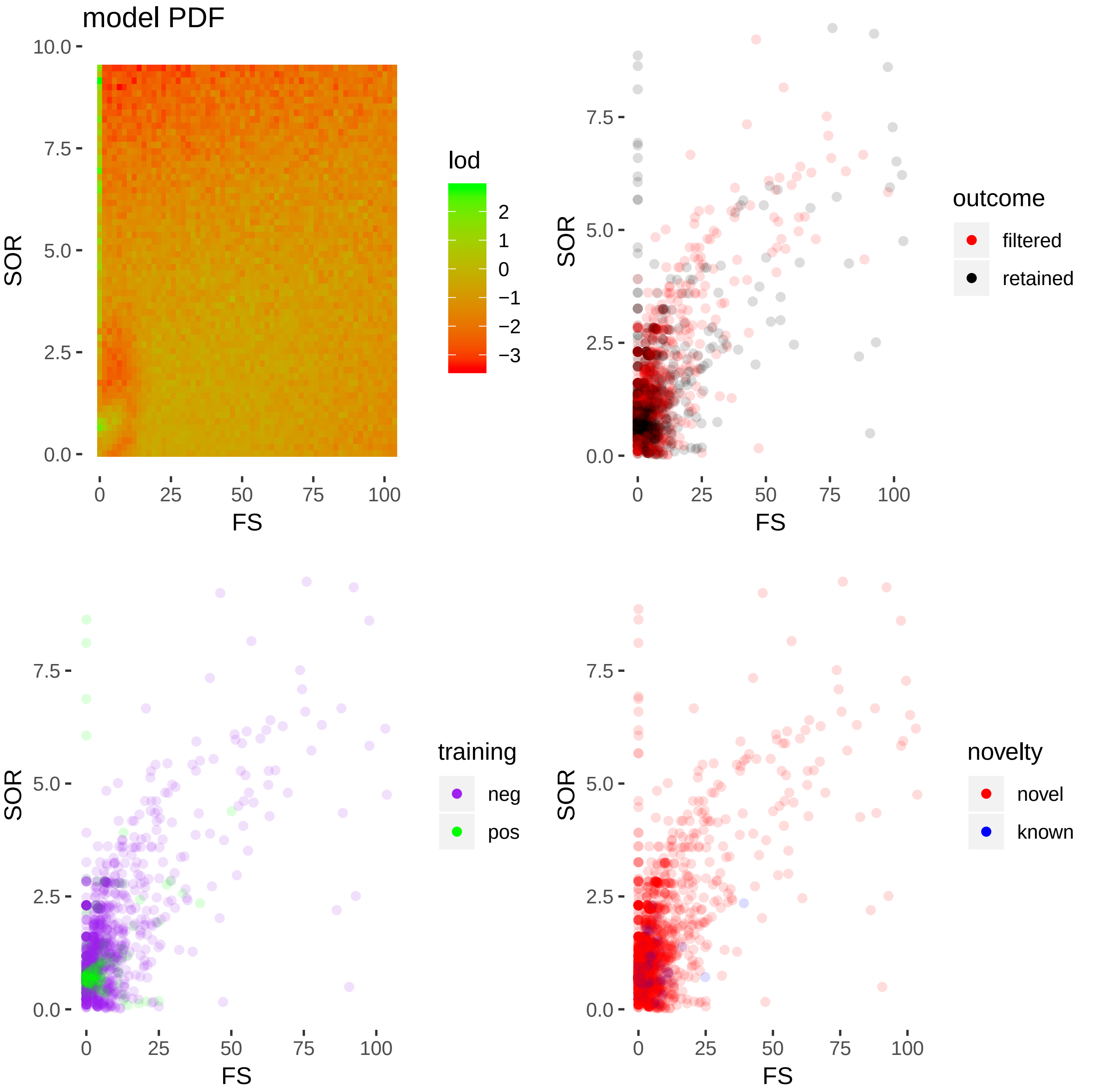
model PDF
lod
outcome
filtered
retained
training
neg
pos
novelty
novel
known
SOR
FS

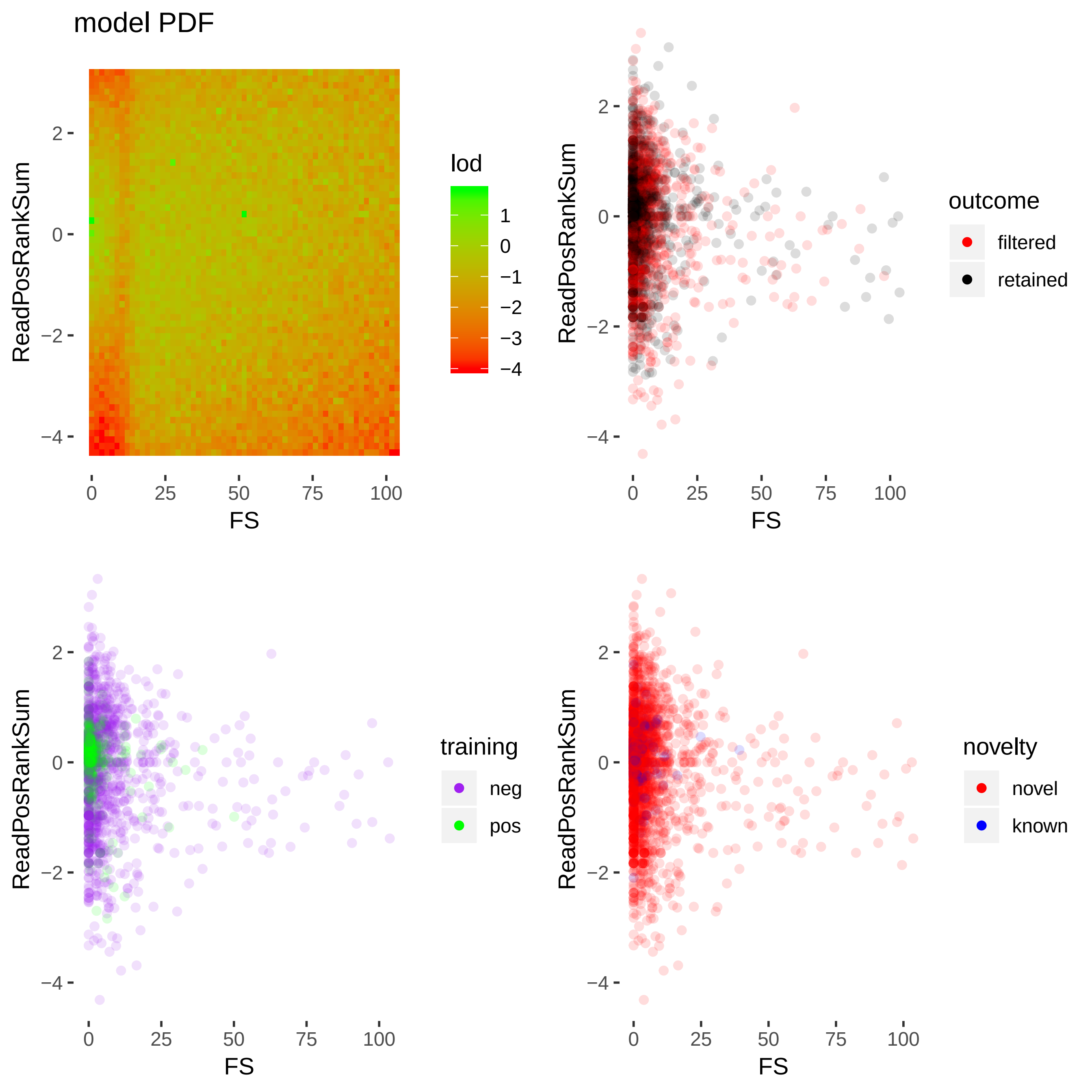

model PDF
lod
ReadPosRankSum
FS
outcome
filtered
retained
training
neg
pos
novelty
novel
known

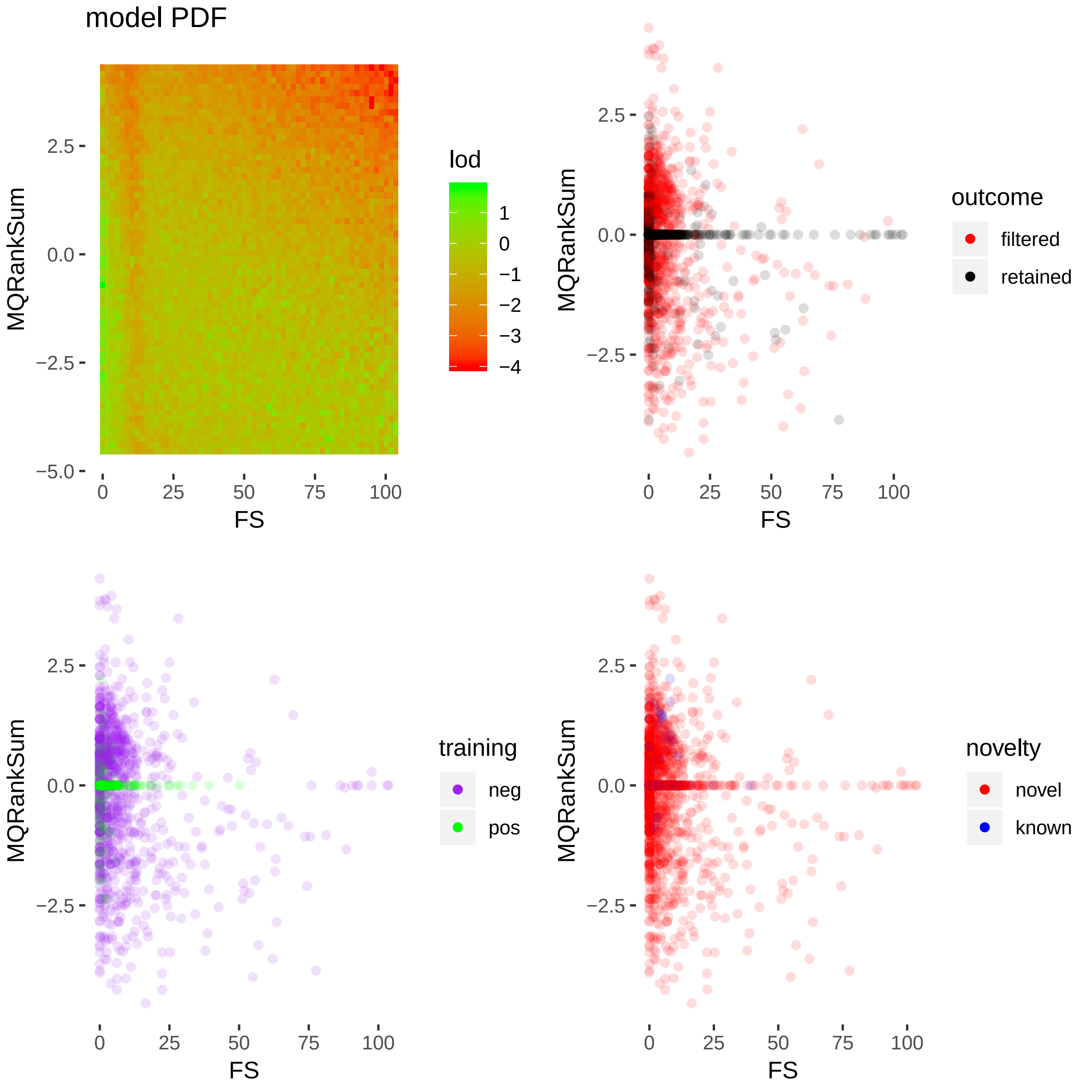

model PDF
lod
1
0
−1
−2
−3
−4
MQRankSum
2.5
0.0
−2.5
−5.0
0
25
50
75
100
FS
outcome
filtered
retained
training
neg
pos
novelty
novel
known

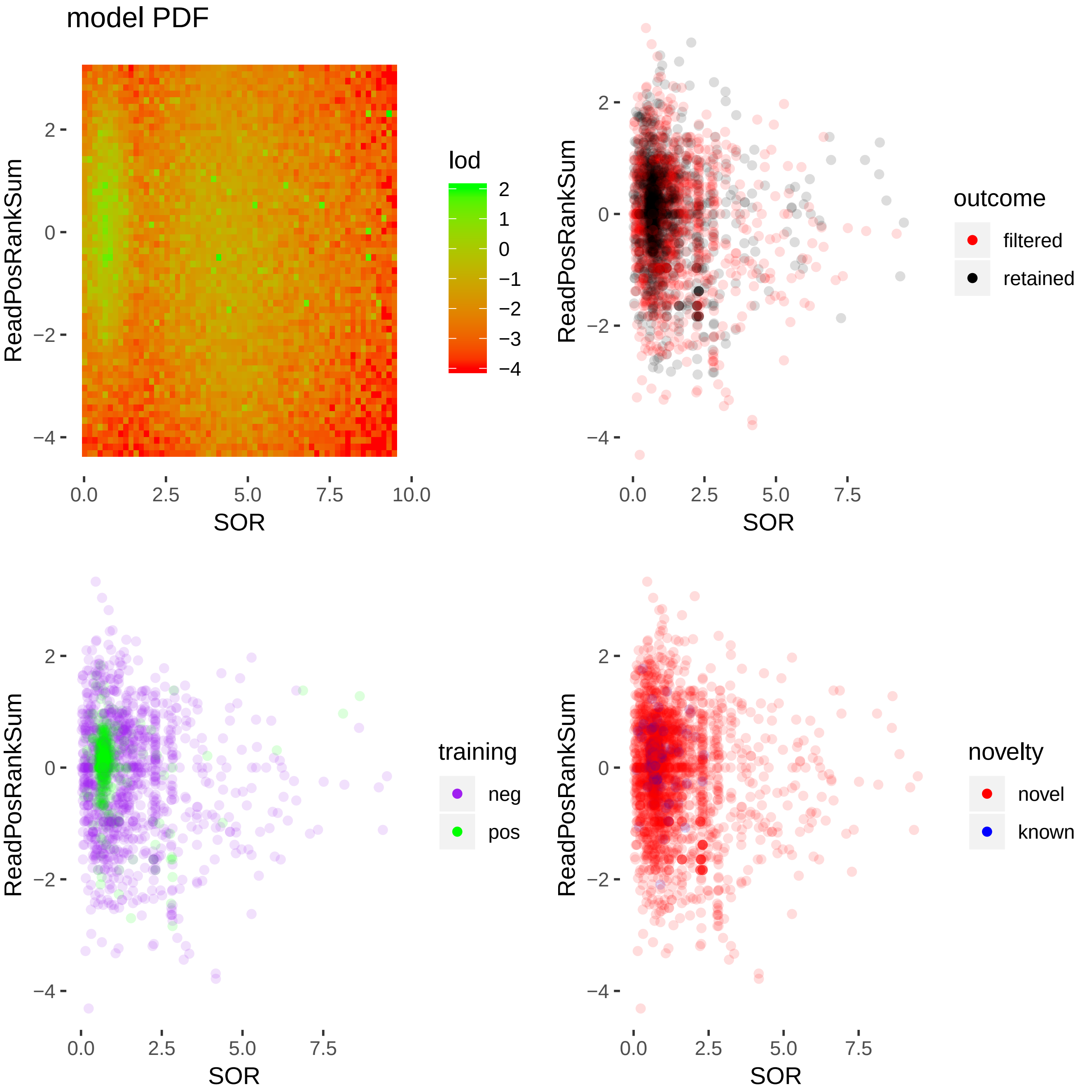
model PDF
lod
2
1
0
−1
−2
−3
−4
ReadPosRankSum
SOR
0.0
2.5
5.0
7.5
10.0
outcome
filtered
retained
training
neg
pos
novelty
novel
known

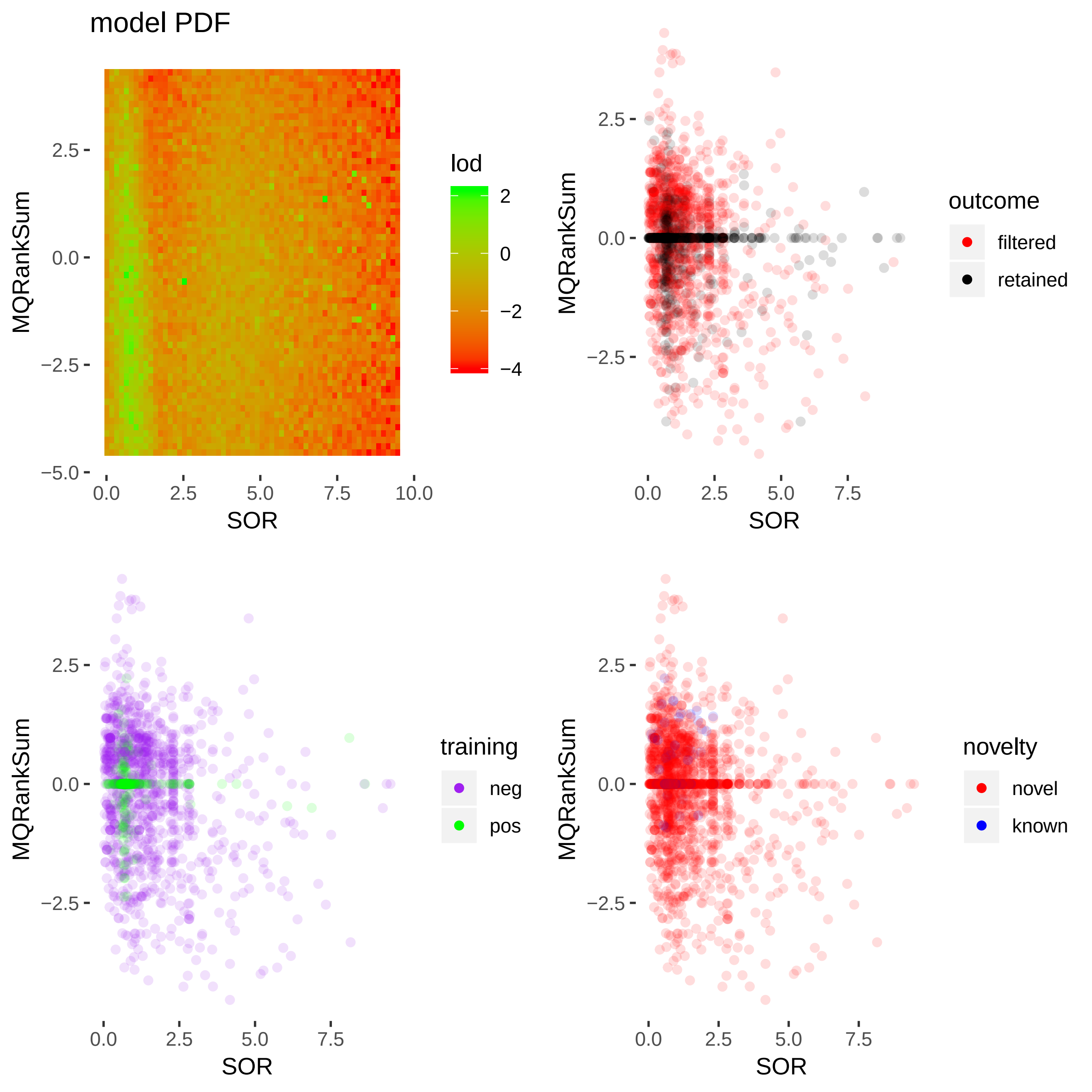

model PDF
MQRankSum
SOR
lod
2
0
−2
−4
2.5
0.0
−2.5
−5.0
5.0
7.5
10.0
outcome
filtered
retained
training
neg
pos
novelty
novel
known

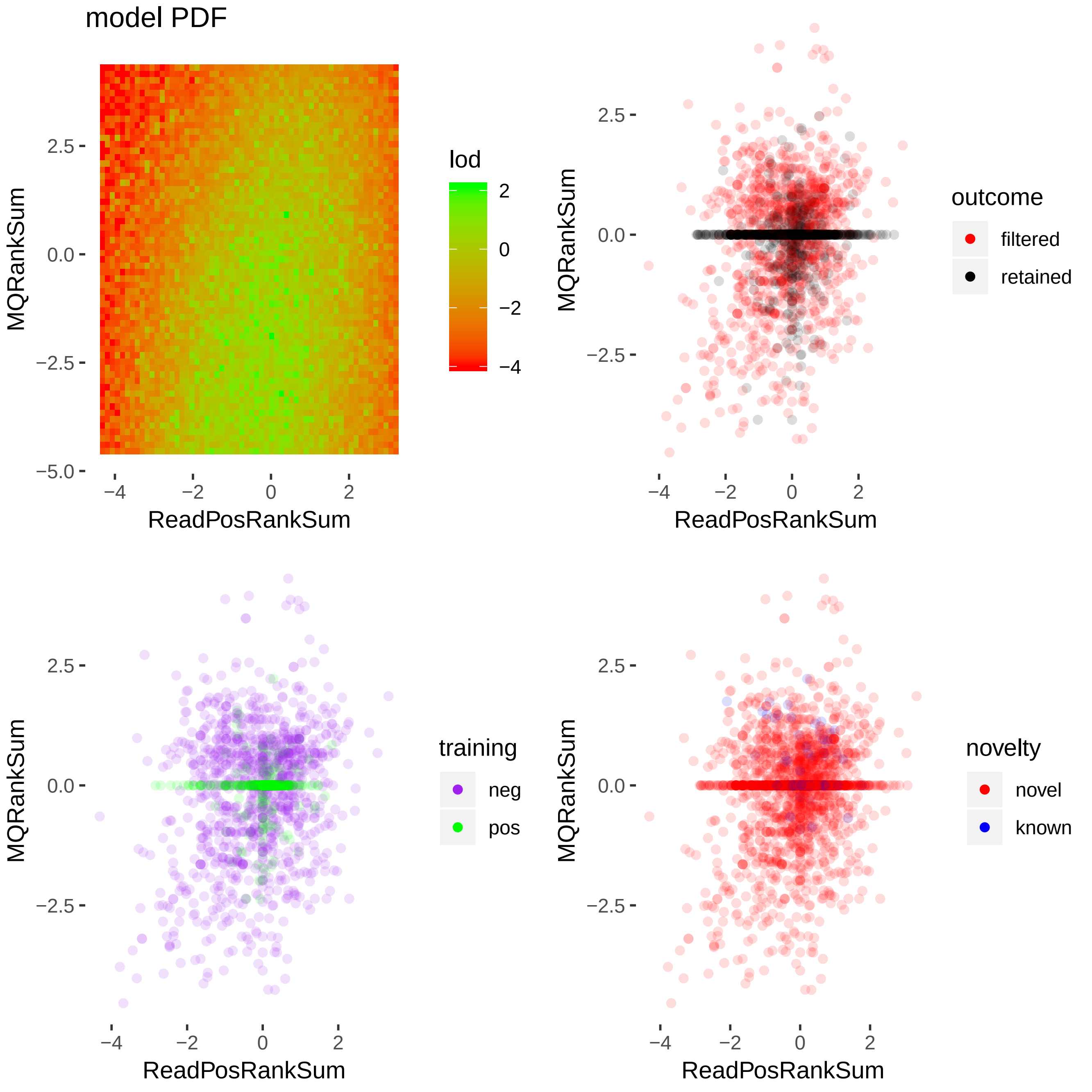
model PDF
lod
2
0
−2
−4
MQRankSum
ReadPosRankSum
2.5
0.0
−2.5
−5.0
−4
−2
0
2
outcome
filtered
retained
training
neg
pos
novelty
novel
known
